# Comparative genomic and phylogenetic analyses of *Crataegus* chloroplast genomes: insights for evolution and identification

**DOI:** 10.3389/fpls.2026.1767012

**Published:** 2026-02-11

**Authors:** Xinyu Sun, Mingqi Cui, Baipeng Zhao, Yu Wang, Xiao Zhang, Yuexue Liu

**Affiliations:** 1 College of Horticulture, Shenyang Agricultural University, Shenyang, China; 2 National Field GeneBank for Hawthorn, Shenyang, China

**Keywords:** biogeography, chloroplast genome, comparative genomics, *Crataegus*, divergence time, phylogenetic relationships

## Abstract

*Crataegus* spp. plants are valuable horticultural crops because of their extensive use in Chinese herbal medications, cosmetics, food production, and other industries. However, the wide variety of species, similar morphological characteristics, inherent hybridization, apomixis, and polyploidy have led to confusion in terms of their taxonomic status. Herein, a total of 18 complete chloroplast genomes including 17 *Crataegus* species and 1 *Mespilus* species were newly sequenced and comprehensively analyzed for comparative genomics and phylogenetic relationships. The 18 chloroplast genomes possessed typical quadripartite structures with lengths from 159,638 to 159,973 bp in size. These chloroplast genomes encode 119–131 genes, including 37 transfer RNA (rRNA) genes, 8 ribosomal RNA (tRNA) genes, and 74–85 protein-coding genes (PCGs). In addition, 23–54 long repeat sequences and 74–87 simple sequence repeats (SSRs) were detected. The examination of Ka/Ks ratios for 18 chloroplast genomes revealed that the *rpoC2* gene was significantly positively selected. Additionally, we identified nine distinct hotspot regions (*infA*, *ndhC*, *pasl*, *rps19*, *ndhC~trnV-UAC*, *psbZ~trnG-UCC*, *rpl33~rps18*, *trnH-GUG~psbA*, and *trnR-UCU~atpA*), and verified that *ndhC~trnV-UAC* might be used as a foundation for subsequent molecular marker studies aimed at identifying *Crataegus* species. Maximum likelihood and Bayesian phylogenetic trees using chloroplast genome sequences consistently revealed genetic relationships among *Crataegus* and *Mespilus* species, and confirmed the taxonomic status of *Crataegus* accessions (GSSZ, JRY, RR2H, RR3H, ZWSZ). The results of divergence time showed that the crown age of C. subg. Crataegus was about 33.487 Ma, and then started to diverge into the C. subg. Americanae and C. subg. Sanguineae around 27.059 Ma. Based on the results of molecular evidence, we speculate that genus *Crataegus* originated earliest from European-derived species within C. subg. Crataegus. Biogeographic and molecular dating analyses suggested that China represented a putative maternal origin of *Crataegus* species. The complete chloroplast genomes of *Crataegus* not only enable the resolution of phylogenetic relationships within the genus but also offer novel insights into chloroplast genome structure variation and evolution. Additionally, the identified divergent DNA regions hold significant utility for species identification and phylogenetic reconstruction in *Crataegus*.

## Introduction

*Crataegus* spp. plants are valuable horticultural crops due to their extensive use in Chinese herbal medications, cosmetics, food production, and other industries (Liu et al., 2020; Liang et al., 2022; Zhang et al., 2025). *Crataegus* spp. plants have been used for centuries as traditional medicines and herbal drugs (Rocchetti et al., 2020). More than 150 substances, such as polysaccharides, phenolics, and flavonoids, have been extracted from its leaves and fruits and have been used to treat hypertension and cardiovascular and cerebrovascular diseases (Tassell et al., 2010; Cloud et al., 2020; Feng et al., 2022). The leaves and flowers *of Crataegus* sp. can also be used to make nanocapsules, which are widely used in the pharmaceutical, cosmetic, and fragrance industries (Esmaeili et al., 2013). Furthermore, the xylan in *Crataegus* kernels can be converted into products such as xylose and xylooligosaccharides (Liu et al., 2020).

In the northern temperate zones of North America, Europe, and Asia, *Crataegus* species, which are members of the Rosaceae family, are extensively distributed (Dong and Li, 2015). Because of its natural hybridization, apomixis, polyploidy, and similar morphological traits, *Crataegus* is a challenging species to identify. The genus *Crataegus* contains more than 200 species (Phipps et al., 1990; Campbell et al., 2007; Benli et al., 2008). China is the primary origin of both cultivated and wild *Crataegus* species, with eighteen species and six varieties (Zhao and Feng, 1996). However, some researchers claim that there are twenty species and seven varieties of Chinese *Crataegus* (Dong and Li, 2015).

**Figure 1:**
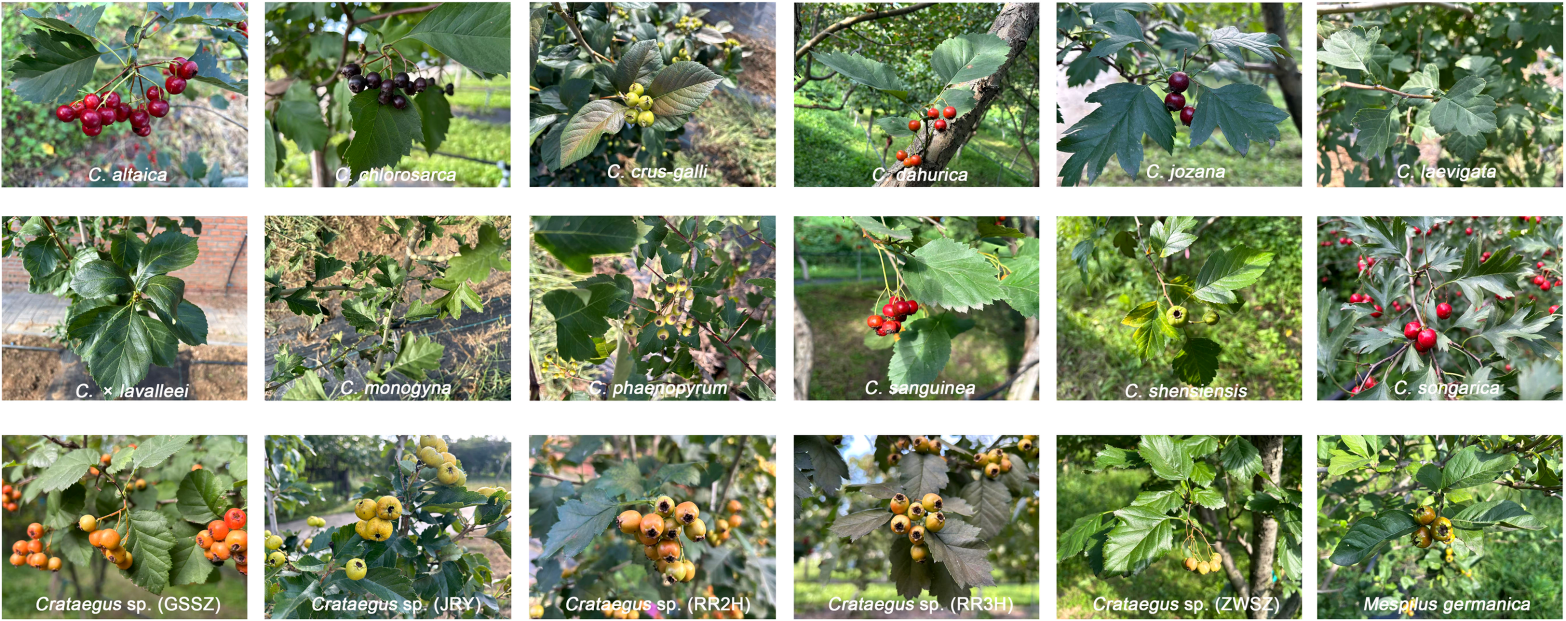
Morphological characteristics of seventeen *Crataegus* and one *Mespilus* species.

Morphological traits have been identified as significant indices in the identification of *Crataegus* species (Dickinson et al., 1996). Nonetheless, the conventional classification of *Crataegus* according to morphological characteristics has been contested and is influenced by environmental factors (Gosler et al., 1994). Most *Crataegus* plants native to China have corymbs and contain a single white flower. Thus, the classification of *Crataegus* plants is based mainly on the morphological characteristics of the leaves and fruits. For example, both *C. chlorosarca* and *C. jozana* have black and spherical fruits (**Figure 1**). The fruits of *C. dahurica* and *C. sanguinea* share similar morphological traits: both are subspherical and exhibit an orange-red or orange-yellow color. Their leaf morphologies are also comparable (**Figure 1**). In addition, the taxonomic status of the *Crataegus* accessions (ZWSZ, GSSZ, RR3H and RR5H) could not be determined based on the morphological traits of leaf and fruit; in the phylogenetic tree constructed using nuclear Simple sequence repeats (nSSR) markers and specific locus amplified fragment sequencing (SLAF-seq) data, these accessions showed a closer genetic relationship with *C. maximowiczii* and *C. sanguinea* (Du et al., 2019). Therefore, clarifying the phylogenetic and taxonomic relationships among *Crataegus* species and establishing a standardized identification system are of great significance for related research and applications.

Molecular markers are among the most accurate ways to determine the genetic links of entire plant populations (Güney et al., 2018). Several innovative DNA-based markers have been rapidly created for recognizing the *Crataegus* genome and investigating genetic variability within and between wild landraces of this species. These marker techniques include simple sequence repeats (SSRs) (Khiari et al., 2015), intersimple sequence repeats (ISSRs) (Sheng et al., 2017; Tunç et al., 2025), randomly amplified polymorphic DNA (RAPD) (Yilmaz et al., 2010), and start codon targeted polymorphism (SCoT) (Sagbas et al., 2023; Mohammed et al., 2025). Owing to inadequate information and coverage, these molecular markers may not be sufficient for high-resolution genetic studies of *Crataegus* plants.

Chloroplasts have lived on Earth for billions of years by offering carbohydrates, amino acids, lipids and O_2_ to humans through the process of photosynthesis (Daniell et al., 2021). Following the discovery of organellar DNA, the first complete chloroplast genome was published in 1986 (Shinozaki et al., 1986). Notably, the chloroplast genome features a low mutation rate (Green, 2011), as its synonymous nucleotide substitution rate (dS) in angiosperms is three-fold higher than that of plant mitochondrial DNA; in contrast, the structural variability of the chloroplast genome is substantially lower than that of both mitochondrial and nuclear genomes (Wang et al., 2024). Structural variations (e.g., insertions, inversions, deletions) in chloroplast genomes modulate genome size and gene content through processes including gene duplication and pseudogenization (Daniell et al., 2016; Qin et al., 2025). These features have made the chloroplast genome become the primary resource for studies into plant phylogeny and evolution (Cauz-Santos, 2025). As next-generation sequencing technology has advances, an increasing number of researchers are utilizing chloroplast genome data to elucidate the evolutionary relationships among various plant species (Wu et al., 2024; Jiang et al., 2025; Li et al., 2025a, 2025, 2025; Xia et al., 2025; Yan et al., 2025).

Owing to frequent hybridization, parthenogenesis and other factors, the classification of subfamilies, tribes and genera of Rosaceae plants remains controversial. The phylogenetic tree of 79 genera and 132 species of Rosaceae, including two *Crataegus* species (*C. pinnatifida* var. *major*, *C. chungtienensis*) was reconstructed via the chloroplast phylogenomic method, and the phylogenetic relationships among its subfamilies, tribes and genera were successfully analyzed (Zhang et al., 2017). Recent studies proposed a comprehensive subgeneric classification of *Crataegus*, including five subgenera: C. subg. Crataegus, C. subg. Americanae El Gazzar, C. subg. Brevispinae (Beadle) Ufimov & T. A. Dickinson, C. subg. Mespilus (L.) Ufimov & T. A. Dickinson, and C. subg. Sanguineae Ufimov (Ufimov and Dickinson, 2020). Chinese *Crataegus* species are classified into two subgenera: C. subg. Crataegus and C. subg. Sanguineae (Gu et al., 2003). Chloroplast genome sequencing has been completed for several *Crataegus* plants native to China, including *C. scabrifolia*, *C. chungtienensis*, and *C. oresbia* (Wu et al., 2022), *C. pinnatifida* var. *major* (Wu et al., 2021), *C. bretschneideri*, *C. maximowiczii*, *C. maximowiczii* var. *ninganensis* (Zhang et al., 2022), *C. rhipidophylla* from Europe and *C. mollis* from North America (Liu et al., 2022). The comprehensive chloroplast genome data will enhance comparative genomic research and serve as a valuable tool to clarify the interspecific relationships of *Crataegus* species worldwide.

In this study, we selected 17 *Crataegus* species (encompassing 7 identified species and 5 *Crataegus* sp. taxa indigenous to China, 3 North American species, and 2 European species) as well as one *Mespilus* species, aiming to explore the structural variability and genetic diversity of chloroplast genomes from a comparative genomic perspective. To this end, we systematically integrated analyses of relative synonymous codon usage (RSCU), repetitive sequence identification, and selective pressure evaluation to comprehensively characterize the chloroplast genomic features of *Crataegus* species. Specifically, we screened and experimentally validated highly conserved intergenic regions as universal DNA barcodes for species discrimination. In addition, we accurately estimated the species divergence times, reconstructed the patterns of their ancestral geographic distribution, and thereby inferred the evolutionary pathways of *Crataegus* species. Collectively, our results will provide comprehensive genetic and evolutionary insights into these genera, and offering valuable tools for future taxonomic, ecological, and conservation research.

## Materials and methods

### Plant materials and chloroplast genome sequencing

Eighteen individuals of *Crataegus* and *Mespilus* species, including *C. altaica, C. chlorosarca, C. crus-galli, C. dahurica, C. jozana, C. laevigata, C.× lavalleei, C. monogyna, C. phaenopyrum, C. sanguinea, C. shensiensis, C. songarica, Crataegus* sp. (GSSZ, JRY, RR2H, RR3H, ZWSZ), and *M. germanica* were cultivated at the National Field Genebank for Hawthorn, Shenyang, Liaoning Province, China (41°84′N, 123°56′E) (**Supplementary Table S1**). Fresh and healthy leaves were collected and stored at –80 °C for chloroplast genome sequencing. The chloroplast DNA from these leaves was sequenced by Nanjing Genepioneer Biotechnologies (Nanjing, China) via an Illumina NovaSeq 6000 system in paired-end (2 × 150 bp) sequencing mode. The processing of raw sequencing data was based on previous research (Zhang et al., 2022). After the quality control process, high quality reads (clean data) were obtained and stored in the FASTQ format. 51 individuals of *Crataegus* were also cultivated at the National Field Genebank for Hawthorn (**Supplementary Table S2**). Fresh and healthy leaves were collected and stored at –80 °C for DNA extraction and candidate DNA barcode sequencing.

### Assembly and annotation of the chloroplast genome

*De novo* assembly of the chloroplast genome was performed using GetOrganelle v1.7.7.1 with gradient k-mer sizes (55, 87, and 121) to balance assembly sensitivity and accuracy (Jin et al., 2020). To ensure the reliability of the assembled genome, two quality control (QC) steps were conducted: first, sequencing reads were mapped back to the assembled genome to calculate key metrics (e.g., genome coverage and insert size; **Supplementary Table S1**); second, the assembled genome was compared against the reference sequence *C. maximowiczii* (GenBank accession No. NC065485) for further validation.

The GeSeq web service (Tillich et al., 2017) was used to annotate the chloroplast genome. BLAST was performed on the 18 assembled chloroplast genome sequences via the National Center for Biotechnology Information (NCBI) website (https://blast.ncbi.nlm.nih.gov/Blast.cgi), and the most similar annotations were selected as reference genomes. These reference genomes were then uploaded to GeSeq to annotate the 18 chloroplast genomes. The protein-coding genes (PCGs) of the chloroplast genome were annotated via Prodigal v2.6.3 (https://www.github.com/hyattpd/Prodigal), rRNA was predicted via HMMER v3.1b2 (http://www.hmmer.org/), and tRNA was predicted via aragorn v1.2.38 (http://www.ansikte.se/ARAGORN/).

A schematic diagram of the chloroplast genome with the annotation of large single-copy (LSC), small single-copy (SSC), and inverted repeats (IR) regions was obtained via CPGview (Liu et al., 2023). The chloroplast genome of *C. altaica* was compared to the other 17 whole chloroplast genomes of *Crataegus* using CGView Server (Grant and Stothard, 2008). GC distributions were measured based on GC skew using the equation: GC skew = (G-C)/(G + C). The exact boundaries of the IR/LSC and IR/SSC regions were confirmed by alignment with homologous sequences from other *Crataegus* species. The GC content of each section was calculated via EditSeq (Burland, 2000). The genes on the boundaries of the junction sites of the chloroplast genome were analyzed via IRscope (Amiryousefi et al., 2018).

### Relative synonymous codon usage, repeat structure, and microsatellite analysis

PhyloSuite v1.2.2 (Wuhan, China) (Xiang et al., 2023) was used to screen the protein coding genes (PCGs) of 18 *Crataegus* and *Mespilus* chloroplast genomes and to calculate codon preference, which was obtained from the actual frequency of codon use to the theoretical frequency. The calculation method of RSCU was based on a previous study (Zhang et al., 2024). The visualization of codon usage bias was platformed by interactive tool https://pcg-lab.shinyapps.io/RSCU-Plot/.

Repeat structures, including forward, reverse, complement and palindromic repeats within the 49 chloroplast genomes (18 newly sequenced chloroplast genomes, 26 *Crataegus* chloroplast genome datasets retrieved from GenBank in the **Supplementary Table S3**, and **5***Amelanchier* species (MN068257, MN068255, MK920297, MN068262, MK920292) were identified via REPuter (Kurtz and Schleiermacher, 1999). The REPuter parameters were set a minimal repeat size of ≥ 30 bp and a Hamming distance of 3 (90% or greater sequence identity) (Zhang et al., 2022). Tandem repeats were screened via the online program Tandem Repeats Finder v4.07 b (Benson, 1999), and the alignment parameters match, mismatch, and indels were set to 2, 7, and 7, respectively. The minimum alignment scores to report repeats and maximum period size were 70 bp and 500 bp, respectively. Otherwise, single sequence repeats (SSRs) within 49 chloroplast genomes were detected via MISA-web (Beier et al., 2017). When the SSR motif length was 1, 2, 3, 4, 5, and 6, the minimum numbers of repeats in the SSR search parameters were 10, 5, 4, 3, 3, and 3, respectively. The maximum sequence length between two SSRs for registration as a compound SSR was 100 bp.

### Sequence divergence and selective pressure analyses

The 18 newly sequencing chloroplast whole-genome sequences were visualized via the mVISTA online software. PhyloSuite v1.2.2 was used to perform the genomes alignments. Concatenated datasets of chloroplast PCGs and intergenetic regions were constructed separately. The frequencies of nonsynonymous (Ka) and synonymous (Ks) substitutions and the Ka/Ks ratio for each PCG generated from 18 *Crataegus* and *Mespilus* chloroplast genomes were calculated via the software KaKs_Calculator v3.0 (Zhang et al., 2006).

DNA polymorphism analyses (sliding-window analyses) were performed via DnaSP v6 (Rozas et al., 2017) to determine the nucleotide diversity (*Pi*) of complete chloroplast genomes, PCGs, and intergenetic regions. The window length was set to 600 bp, with a step size of 200 bp for complete chloroplast genomes.

### Candidate DNA barcode screening and sequencing

Based on the results of DNA polymorphism analyses, PhyloSuite v1.2.2 was employed to extract the three intergenetic regions with the highest nucleotide diversity from the chloroplast genomes of 44 *Crataegus* and *Mespilus* species. Among these sequences, the intergenic region (*ndhC_trnV-UAC*), which was present in all 44 chloroplast genomes, was selected for sequencing analysis. We amplified the *ndhC_trnV-UAC* sequences of 51 individuals of *Crataegus*. The primers were F-5’-AGACGTACTCCTATTAATG-3’, and R-5’-AAACCTAAAAATTCAAAT-3’. PCR amplification was performed in a reaction mixture with a final volume of 20 μL consisting of 1 μL of template DNA (50 ng), 10 μL of Takara *Ex*Taq^®^ (RR001A), and 2 μL of primers. The PCR conditions were as follows: initial denaturation at 94 °C for 3 min; followed by 35 cycles of 30 s at 94 °C, 30 s at 55 °C, 1 min at 72 °C; and a final extension of 5 min at 72 °C. PCR amplification was carried out in a thermal cycler (Applied Biosystems, USA). PCR products submitted to Sangon Biotech (Co., Ltd., Shanghai, China) for sequencing. All sequences were aligned using MAFFT v7 with the FFT-NS-2 module. The IQ-TREE module in PhyloSuite was used to build a maximum likelihood tree under the TVM+F+R2 model with 5,000 ultrafast bootstraps. The maximum likelihood (ML) trees were visualized via using FigTree v1.4.4 (http://tree.bio.ed.ac.uk/software/figtree/).

### Phylogenetic analyses, divergence time estimation and ancestral area reconstruction

The complete chloroplast genomes of total 49 *Crataegus*, *Mespilus*, and *Amelanchier* species were compared and aligned. The IQ-TREE module v2.2.0 (Minh et al., 2020) in PhyloSuite was used to build a maximum likelihood tree under the TVM+F+R2 model with 5,000 ultrafast bootstraps. The MrBayes module v3.2.7 (Ronquist et al., 2012) under the partition model (nst = 6, rates = invgamma, statefreqpr = Dirichlet (1,1,1,1)) was used to build a Bayesian inference tree. The Bayesian inference (BI) and maximum likelihood (ML) trees were visualized via FigTree v1.4.4 (http://tree.bio.ed.ac.uk/software/figtree/).

The divergence times of total 49 *Crataegus*, *Mespilus*, and *Amelanchier* species were estimated by BEAST v1.10.4 (Suchard et al., 2018). We assigned the fossils to stem *Amelanchier* with a minimum age of 33.9 Ma and a mean and standard deviation of 0.5, which was treated as the calibration constraint according to published article (Xiang et al., 2017). The GTR nucleotide substitution model and the prior tree Yule model were selected with an uncorrelated relaxed clock. Each MCMC run had a chain length of 1,000,000, with sampling every 1,000 steps. Tracer (http://beast.community/tracer) was used to read the ESS and trace value of logged statistics to access the results. The divergence time was subsequently accessed via the Tree-annotator program of BEAST2. The settings used were as follows: burn-in percentage = 50, posterior probability limit = 0.1, target tree type = maximum clade credibility tree, and node height = mean height.

Ancestral area reconstruction and assessment of geographic diversification patterns within *Crataegus* was conducted using BioGeoBEARS (Matzke, 2014) method implemented in RASP v3.2 (Yu et al., 2015). Firstly, the outgroup samples (*Amelanchier*) were deleted from chloroplast genome datasets BEAST MCMC tree utilizing the outgroup-removal tool in the RASP. The model comparison of BioGeoBEARS in RASP was applied to select the best models. A total of six models calculated from the BioGeoBEARS analysis, and BAYAREALIKE+J was the best model (**Supplementary Table S4**). Detailed descriptions of the model parameters can be found in the published article (Matzke, 2022; Vargas et al., 2023). The biogeographic data for species within *Crataegus* was compiled from Plants of the World Online (POWO, https://powo.science.kew.org/), published book and article (Dong and Li, 2015; Meng et al., 2025) Seven biogeographical areas were chosen based on the geographic range: A) South-western China; B) Central Plains and Qinling Mountains of China; C) North-eastern China; D) Mongolia-Siberian region; E) Central and Western Asia; F) Europe; G) North America.

## Results

### Chloroplast genome characteristics of *Crataegus* and *Mespilus* species

**Figure 2:**
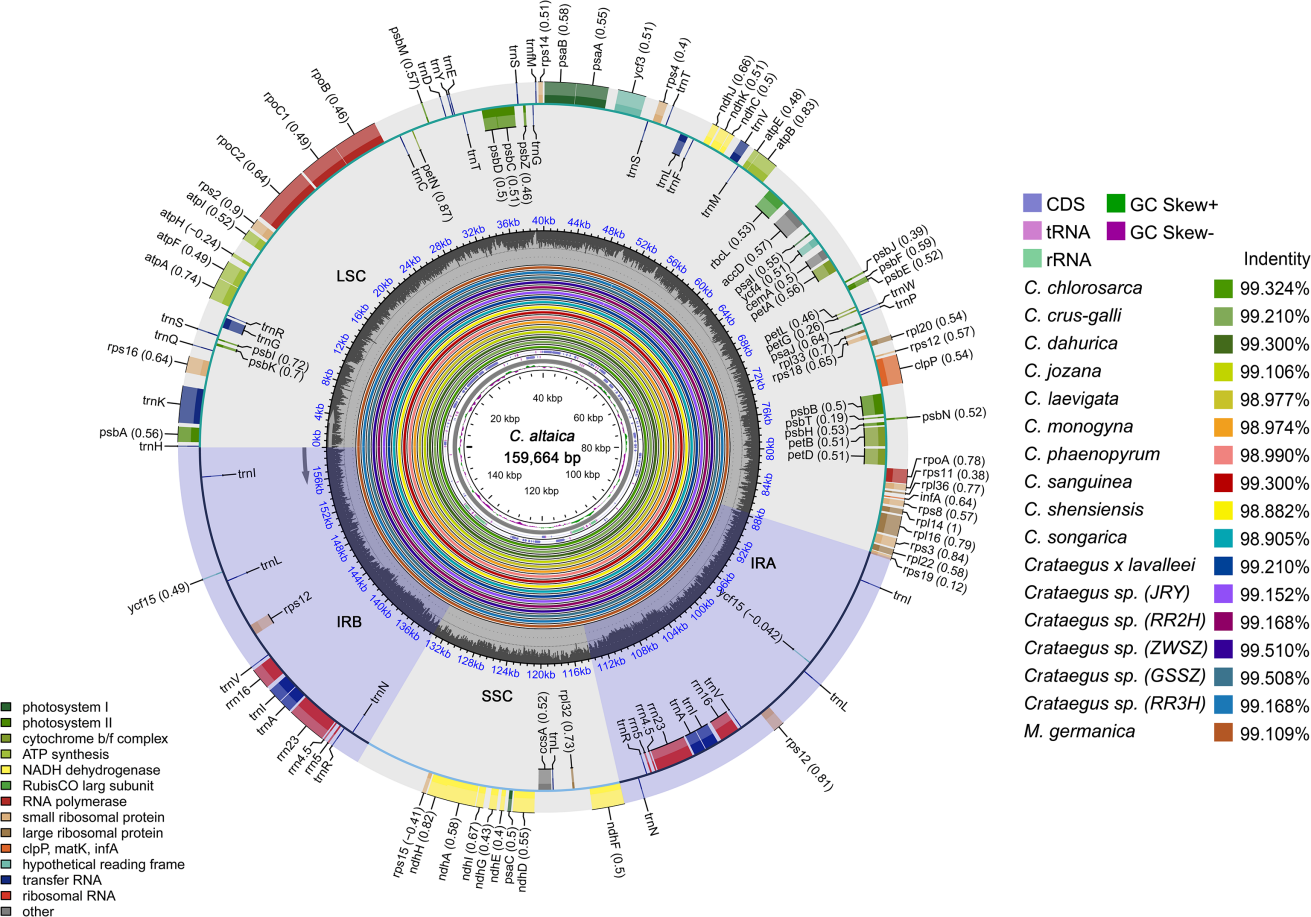
Chloroplast genome map of *C. altaica* (the outermost three rings) and CGView comparison of comparison of 17 complete chloroplast genomes in the *Crataegus* and *Mespilus* species (the inter rings with different colors). Genes shown on the outside of the outermost first ring are transcribed counter-clockwise and on the inside clockwise. Outermost second ring with darker gray corresponds to GC content, whereas outermost third ring with the lighter gray corresponds to AT content of *C. altaica* chloroplast genome by OGDRAW. The gray arrowheads indicate the direction of the genes. LSC, large single copy region; IR, inverted repeat; SSC, small single copy region. The innermost first black ring indicates the chloroplast genome size of *C. altaica.* The innermost second and third rings indicate GC content and GC skews deviations in chloroplast genome of *C. altaica*, respectively: GC skew + indicates G > C, and GC skew- indicates G < C. CGView comparison result of 17 complete chloroplast genomes displayed from innermost fourth color ring to outwards 21th ring in turn: *C.chlorosarca*, *C.crus-galli*, *C. dahurica*, *C. jozana*, *C. laevigata*, *C. monogyna*, *C. phaenopyrum*, *C. sanguinea*, *C. shensiensis*, *C. songarica*, *Crataegus* × *lavalleei*, *Crataegus* sp. (JRY, RR2H, ZWSZ, GSSZ, RR3H), and *M. germanica.* Sequence identity analysis, conducted via CGView, indicate the level of similarity between the chloroplast genome of each *Crataegus* and *Mespilus* species and that of *C. altaica*.

In this study, a total of 18 complete chloroplast genomes of *Crataegus* and *Mespilus* species were analyzed (**Table 1**). The 18 sequenced samples produced 10.11 to 15.55 Gb clean reads each after removal of adapters and low-quality reads (**Table 1**). The 18 complete chloroplast genomes in this study were deposited in the GenBank with accession numbers PX413282 to PX413299 (**Supplementary Table S1**). The complete chloroplast genomes of *Crataegus* ranged from 159,638 (*Crataegus* sp., RR2H and RR3H) to 159,973 bp (*C. phaenopyrum*) in length, with differences of 4~335 bp (**Figure 2**; **Table 1**). The length of the *Mespilus germanica* chloroplast genome was 159,811 bp, similar in length to that of *Crataegus* sp. (GSSZ). The *Crataegus* and *Mespilus* chloroplast genomes contained a typical quadripartite structure containing IRa and IRb regions (26,311~26,396 bp) separated by the LSC (87,665~88,081 bp) and SSC (19,139~19,295 bp) regions. The chloroplast genome characteristics of 12 *Crataegus* species, 5 *Crataegus* sp. plants, and *Mespilus germanica* were similar. The GC contents of these complete chloroplast genomes ranged from 36.59%~36.65%, 34.29%~34.40% in the LSC region, 30.32%~30.56% in the SSC region, and 42.63%~45.62% in the IR region, revealing high similarity among different *Crataegus* and *Mespilus* plants.

**Table 1:** A comparison of 18 chloroplast genomes.

Individuals	Genome size (bp)	LSC size (bp)	SSC size (bp)	IR size (bp)	Number of total genes	Protein coding genes	tRNA genes	rRNA genes	Duplicated genes in IR	GC content (%)	GC content (%)		
											LSC	SSC	IR
*C. altaica*	159664	87665	19245	26377	119 (105)	74 (72)	37 (30)	8 (4)	16	36.63	34.37	30.42	42.65
*C. chlorosarca*	159782	87744	19270	26384	120 (106)	75 (73)	37 (30)	8 (4)	16	36.61	34.36	30.38	42.64
*C. crus-galli*	159771	87806	19235	26365	130 (112)	85 (79)	37 (30)	8 (4)	16	36.61	34.33	30.42	42.67
*C. dahurica*	159858	87850	19240	26384	120 (106)	75 (73)	37 (30)	8 (4)	16	36.60	34.33	30.43	42.63
*C. jozana*	159712	87784	19160	26384	132 (112)	87 (79)	37 (30)	8 (4)	16	36.64	34.37	30.55	45.62
*C. laevigata*	159826	87841	19217	26384	120 (106)	75 (73)	37 (30)	8 (4)	16	36.62	34.35	30.46	42.63
*C. × lavalleei*	159771	87806	19235	26365	131 (113)	85 (79)	37 (30)	8 (4)	16	36.61	34.33	30.42	42.67
*C. monogyna*	159805	87822	19215	26384	120 (106)	75 (73)	37 (30)	8 (4)	16	36.62	34.36	30.43	42.63
*C. phaenopyrum*	159973	88081	19230	26331	130 (112)	85 (79)	37 (30)	8 (4)	16	36.59	34.29	30.47	42.67
*C. sanguinea*	159858	87850	19240	26384	120 (106)	75 (73)	37 (30)	8 (4)	16	36.60	34.33	30.43	42.63
*C. shensiensis*	159807	87779	19262	26383	120 (106)	75 (73)	37 (30)	8 (4)	16	36.62	34.37	30.42	42.64
*C. songarica*	159882	87891	19213	26389	120 (106)	75 (73)	37 (30)	8 (4)	16	36.61	34.33	30.45	42.64
*Mespilus germanica*	159811	87804	19215	26396	128 (112)	83 (78)	37 (30)	8 (4)	16	36.60	34.40	30.50	42.64
*Crataegus* sp. (GSSZ)	159815	87752	19295	26384	120 (106)	75 (73)	37 (30)	8 (4)	16	36.60	34.35	30.32	42.64
*Crataegus* sp. (JRY)	159655	87748	19139	26384	132 (112)	85 (79)	37 (29)	8 (4)	16	36.65	34.38	30.56	42.64
*Crataegus* sp. (RR2H)	159638	87731	19139	26384	132 (112)	85 (79)	37 (29)	8 (4)	16	36.65	34.38	30.56	42.64
*Crataegus* sp. (RR3H)	159638	87731	19139	26384	132 (112)	87 (79)	37 (30)	8 (4)	16	36.65	34.38	30.56	42.64
*Crataegus* sp. (ZWSZ)	159814	87752	19294	26384	132 (112)	85 (79)	37 (29)	8 (4)	16	36.60	34.35	30.33	42.64

The numbers in parenthesis indicate the duplicated genes in chloroplast genomes. LSC, large single-copy; SSC, small single-copy; IR, inverted repeats; tRNA, transfer RNA; rRNA, ribosomal RNA.

In general, the complete chloroplast genomes of *Crataegus* and *Mespilus* plants encoded 119~131 genes, including 74~85 PCGs (protein coding genes), 37 tRNA genes, and 8 rRNA genes (**Table 1**). Several PCGs (*ndhB*, *rpl23*, *rpl2*, *rps7*, *ycf2*, and *rps12*), tRNA genes (*trnN-GUU*, *trnR-ACG*, *trnA-UGC*, *trnI-GAU*, *trnV-GAC*, *trnL-CAA*, and *trnI-CAU*), and rRNA genes (*rrn5*, *rrn4.5*, *rrn23*, and *rrn16*) had one duplicated gene. The annotated chloroplast genomes of *Crataegus* and *Mespilus*, including their gene numbers, orders, and names are represented in a circular map (**Figure 2**). Among the 113 unique genes, PCGs (*rps16*, *atpF*, *rpoC1*, *petB*, *petD*, *rpl16*, *rpl2*, *ndhB*, and *ndhA*) and tRNA genes (*trnK-UUU*, *trnG-GCC*, *trnL-UAA*, *trnV-UAC*, *trnI-GAU*, and *trnA-UGC*) had one intron; PCGs (*rps12*, *clpP* and *ycf3*) contained two introns (**Table 2**). The locations and numbers of introns of genes in the *Crataegus* and *Mespilus* chloroplast genomes presented similar features. Through CGView-based sequence identity analysis, the similarity levels between the chloroplast genomes of individual *Crataegus* and *Mespilus* species and *C. altaica* were characterized (**Figure 2**). The results of this analysis showed that the sequence identity ranged from 98.882% to 99.510%, indicating that these 18 chloroplast genomes were relatively conserved and exhibit high sequence similarity.

**Table 2:** List of genes encoded in chloroplast genome.

Category of genes	Gene group	Gene name
Photosynthesis	Photosystem I	*psaA, psaB, psaC, psaI, psaJ*
	Photosystem II	*psbA, psbB, psbC, psbD, psbE, psbF, psbH, psbI, psbJ, psbK, psbL, psbM, psbN, psbT, psbZ*
	NADH dehydrogenase	*^a^ndhA, ^a,c^ndhB, ndhC, ndhD, ndhE, ndhF, ndhG, ndhH, ndhI, ndhJ, ndhK*
	Cytochrome b/f complex	petA, ^a^petB*, ^a^petD*, petG, petL, petN
	ATP synthase	*atpA, atpB, atpE, ^a^atpF, atpH, atpI*
	Rubisco (Large submit)	*rbcL*
Self-replication	Ribosome (Large submit)	*rpl14, ^a^rpl16, ^a,c^rpl2, rpl20, rpl22, ^c^rpl23, rpl32, rpl33, rpl36*
	Ribosome (Small subunit)	*rps11, ^b,c^rps12, rps14, rps15, ^a^rps16, rps18, rps19, rps2, rps3, rps4, ^c^rps7, rps8*
	RNA polymerase	*rpoA, rpoB, ^a^rpoC1, rpoC2*
	Ribosomal RNAs	*^c^rrn16, ^c^rrn23, ^c^rrn4.5, ^c^rrn5*
	Transfer RNAs	*^a,c^trnA-UGC, trnC-GCA, trnD-GUC, trnE-UUC, trnF-GAA, ^a^trnG-GCC, trnG-UCC, trnH-GUG, ^c^trnI-CAU, ^a,c^trnI-GAU, ^a^trnK-UUU, ^c^trnL-CAA, ^a^trnL-UAA, trnL-UAG, trnM-CAU, ^c^trnN-GUU, trnP-UGG, trnQ-UUG, ^c^trnR-ACG, trnR-UCU, trnS-GCU, trnS-GGA, trnS-UGA, trnT-GGU, trnT-UGU, ^c^trnV-GAC, ^a^trnV-UAC, trnW-CCA, trnY-GUA, trnfM-CAU*
Other genes	Maturase	*matK*
	Protease	*^b^clpP*
	Envelope membrane protein	*cemA*
	Acetyl-CoA carboxylase	*accD*
	c-type cytochrome synthesis gene	*ccsA*
	Translation initiation factor	*infA*
Function-unknown genes	Conserved open reading frames	*^#^ycf1, ycf1, ^c^ycf2, ^b^ycf3, ycf4*

a, Gene with one introns; b, Gene with two introns; c, Number of copies of multi-copy genes; #, Gene: Pseudo gene.

### Border region variations in the chloroplast genomes

**Figure 3:**
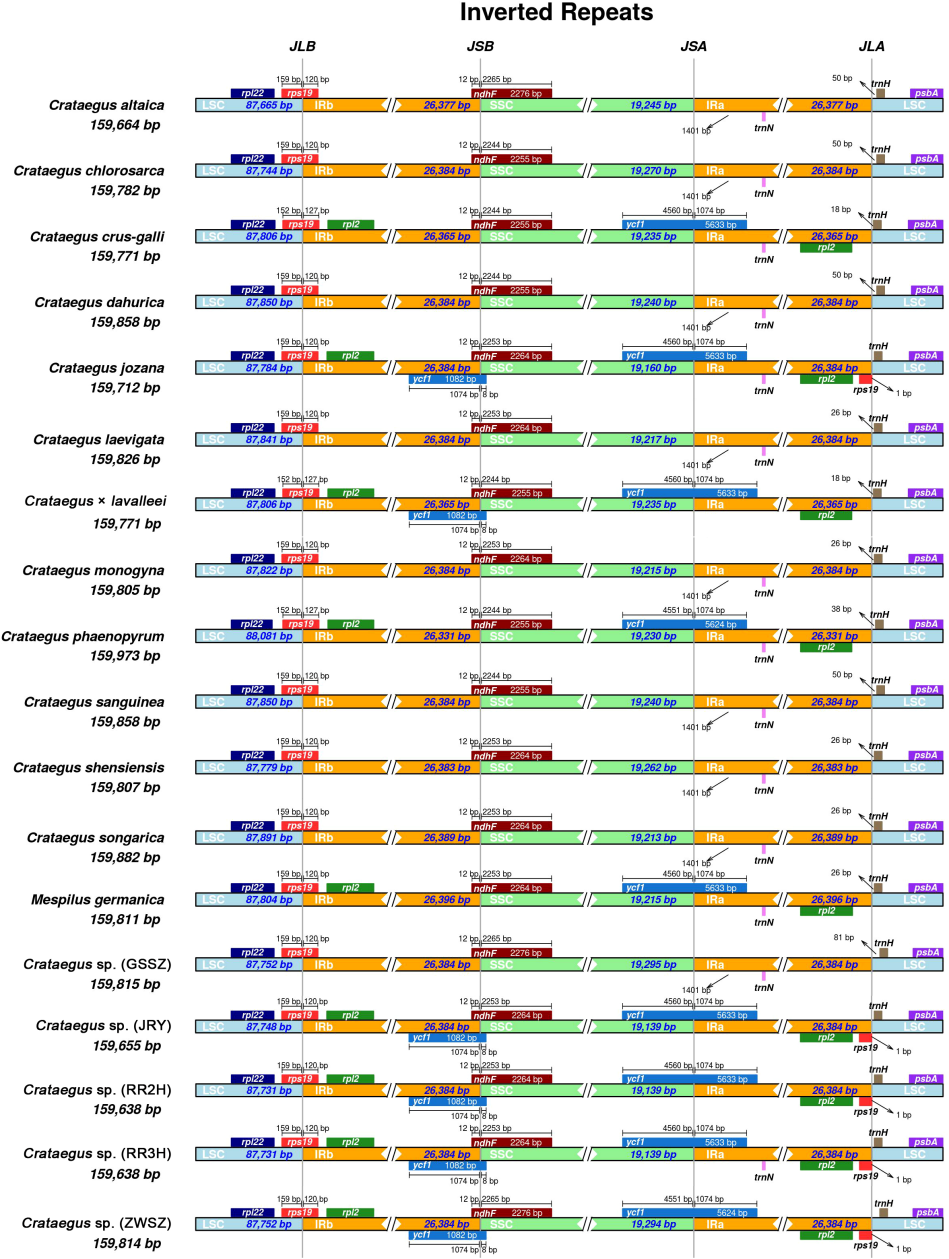
Comparisons of the borders of large single-copy (LSC), small single-copy (SSC), and inverted repeats (IR) regions among 18 chloroplast genomes.

IRscope was used to visualize the genes on the boundaries of the junction sites of the *Crataegus* and *Mespilus* chloroplast genomes. The adjacent genes and border regions of 18 *Crataegus* and *Mespilus* species were analyzed, and *C. kansuensis* (MF784433) was used as the reference (**Figure 3**). In general, the genomic structure was relatively conserved. However, 18 *Crataegus* and *Mespilus* chloroplast genomes presented variations at the LSC/IRb, IRb/SSC, and IRa/LSC borders. The LSC/IRb regions of nine *Crataegus* and *Mespilus* species contained the *rpl2* gene, which is located after the r*ps19* gene. The IRb/SSC regions of the 6 *Crataegus* species contained the *ycf1* gene.

The length of the *ndhf* gene spans the IRb/SSC regions, and its length in the SSC region was differs among *Crataegus* and *Mespilus* species (2,255 bp to 2,276 bp). The *trnH* gene is closely located 18 bp from the junction in *C. crus-galli* and 38 bp from the junction in *C. phaenopyrum.* In other *Crataegus* species, the *trnH* gene is located near the junction at distances ranging from 18~81 bp. The *yfc1* gene of 9 *Crataegus* and *Mespilus* species crossed the SSC/IRa junction, extending the same length in the SSC region (4,551 bp or 4,560 bp) and IRa region (1,074 bp). The variations in these boundary regions resulted in differences in the lengths of the *Crataegus* and *Mespilus* chloroplast genomes and their LSC, IRs, and SSC regions.

### Codon use analysis of protein coding genes

Protein coding genes (PCGs) of the *Crataegus* and *Mespilus* chloroplast genomes were extracted and subjected to codon use analysis. Among the 18 chloroplast genomes, arginine (Arg) (5.9998%~6.0001%), serine (Ser) (5.9999%~6%), and leucine (Leu) (5.9999%~6.0001%) were the most frequently occurring amino acids. In contrast, tryptophan (Trp) (1%) and methionine (Met) (1%) were identified infrequently. In addition, the relative synonymous codon usage (RSCU) was investigated among the 18 chloroplast genomes. The results revealed that 30 types of codons had RSCU values of more than 1.0 in the PCGs of 18 chloroplast genomes, revealing that they were used more than synonymous codons (**Supplementary Figure S1**; **Supplementary Table S5**).

### Repeat sequence and microsatellite assays

**Figure 4:**
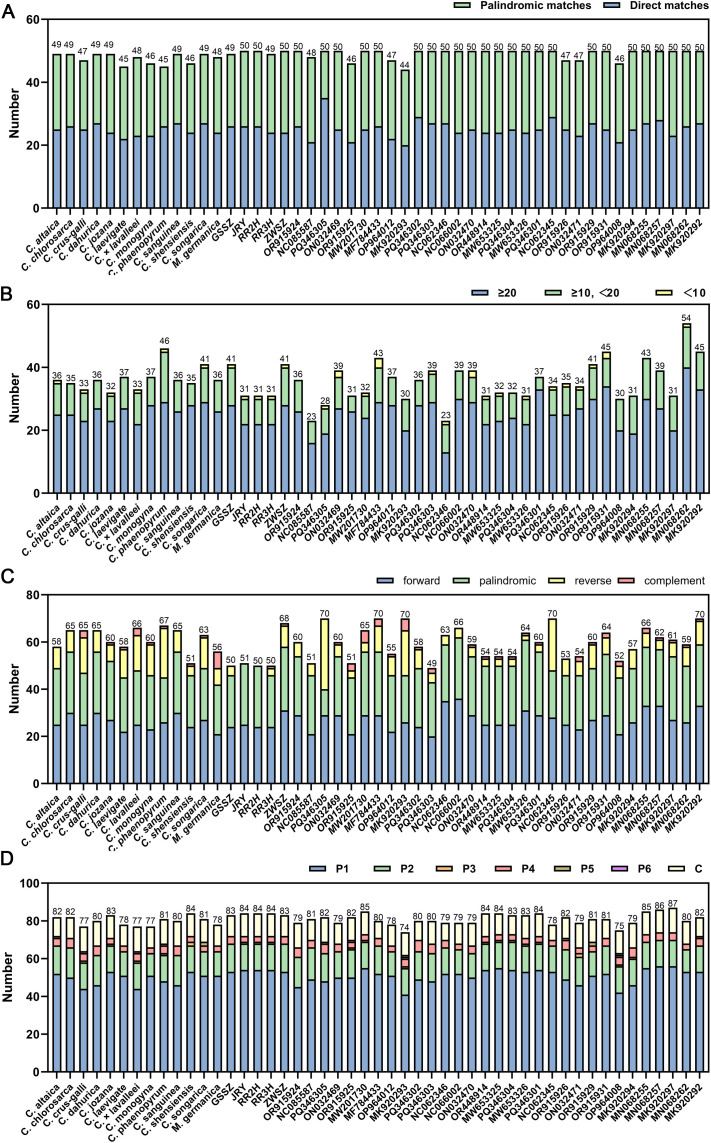
Statistics of repeat elements in 49 chloroplast genomes. **(A)** Number of dispersed repeat sequences; **(B)** number of long tandem repeats; **(C)** number of repeat structures; **(D)** total number of short sequence repeats (SSRs) classified by repeat unit type and repeat unit count. P1, P2, P3, P4, P5, P6, C represent mono-nucleotide, di-nucleotide, tri-nucleotide, tetra-nucleotide, penta-nucleotide, hexa-nucleotide, and compound SSRs, respectively. OR915924, *C*. *altaica*; NC085587, *C*. *aurantia*; PQ346305, *C.* bretschneideri; ON032469, *C. chungtienensis*; OR915925, *C. cuneata*; MW201730, *C. hupehensis*; MF784433, *C. kansuensis*; OP964012, *C. laevigata*; MK920293, *C. marshallii*; PQ346302, *C. maximowiczii*; PQ346303, *C. maximowiczii* var. *ninganensis*; NC062346, *C. mollis*; NC066002, *C. monogyna*; ON032470, *C. oresbia*; OR448914, *C. pinnatifida*; MW653325, *C. pinnatifida*; PQ346304, *C. pinnatifida*; MW653326, *C. pinnatifida* (f) *major*; PQ346301, *C. pinnatifida* (f) *major*; NC062345, *C. rhipidophylla*; OR915926, *C. sanguinea*; ON032471, *C. scabrifolia*; OR915929, *C. songaric*a; OR915931, *C. wilsonii*; OP964008, *C. viridis*; MK920294, *Crataegus* sp.; MN068257, *A*. *cusickii*, MN068255, *A*. *alnifolia*, MK920297, *A*. *ovalis*, MN068262, *A*. *sanguinea*; MK920292, *A*. sp*icata*.

Three types of repeat sequences were analyzed in this study, including dispersed repeats, long tandem repeats, and repeat structure sequences. Dispersed repeat sequences within the 49 chloroplast genomes were identified via VMATCH. A total of 45~50 repeat sequences were present in 49 chloroplast genomes, including 20~35 direct matches and 15~27 palindromic matches in 49 chloroplast genomes (**Figure 4A**). Long tandem repeats were also identified using the following parameters: “2, 7, 7, 80, 10, 70, 500, -f, -d, -m”. In total, 23~54 long tandem repeat sequences were detected among 49 chloroplast genomes (**Figure 4B**). Among these, 0%~ 6.98% were shorter than 10 bp, 10.81%~ 39.13% were between 10 and 20 bp, and 56.52%~ 89.19% were longer than 20 bp. Repeat structures, including forward, reverse, complement and palindromic repeats, within these chloroplast genomes were also identified. In general, 49~70 repeat sequences were explored, including 20~36 forward repeat sequences, 11~30 palindromic repeat sequences, 0~30 reverse repeat sequences, and 0~7 complement repeat sequences (**Figure 4C**). Single sequence repeats (SSRs) within 49 chloroplast genomes were detected (**Figure 4D**; **Supplementary Table S6**). In general, 74~87 SSRs were predicted in these chloroplast genomes. Mono-nucleotides (P1) were the most abundant SSRs in each genome, ranging in quantity from 41 (*C. marshallii*, MK920293) to 56 (*A. cusickii*, MN068257, *A. ovalis*, MK920297).

### Sequence divergence and estimation rate analysis

**Figure 5:**
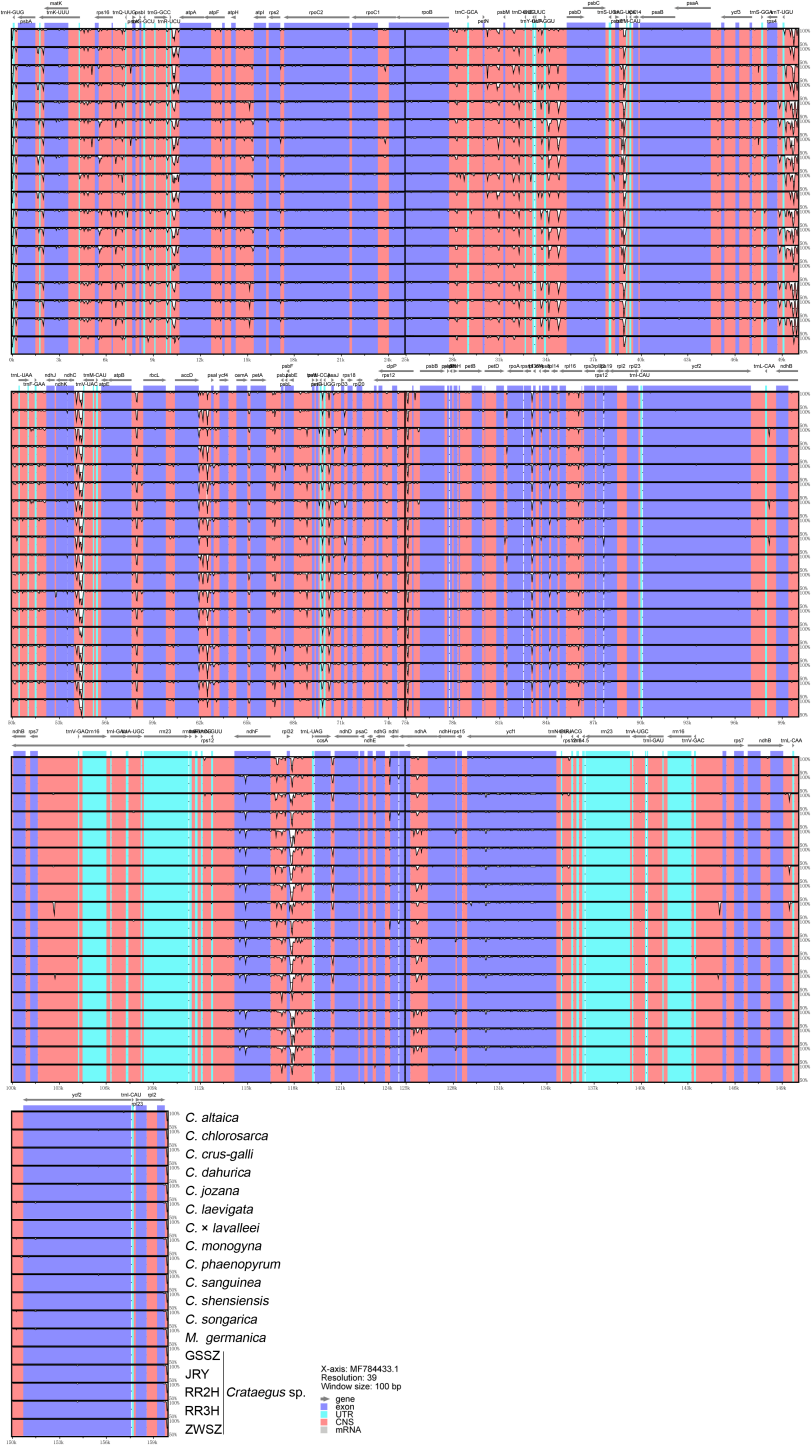
Visualization of the comparison of 18 chloroplast genomes. The horizontal axis represents the coordinates within the chloroplast genome, and the vertical axis indicates the percentage identity, ranging from 50% to 100%. The colors represent different regions: blue for exons, green for introns, and red for intergenetic regions.

The chloroplast genomes of 18 *Crataegus* and *Mespilus* species were compared and analyzed with the chloroplast genome of *C. kansuensis* used as a reference (**Figure 5**). The results revealed that the chloroplast genomes of the 18 *Crataegus* and *Mespilus* species presented minimal interspecies variation. The exon and UTR regions (shown in blue) presented the highest level of conservation, particularly in *ycf2*, *rrn23*, and *rrn16*. In contrast, intergenetic regions presented the greatest variability, with rapid changes in regions such as *trnR-UCU~atpA*, *trnT-UCU~trnL-UAA*, *ndhC~trnV-UAC*, and *rpl32~trnL-UAG.*

**Figure 6:**
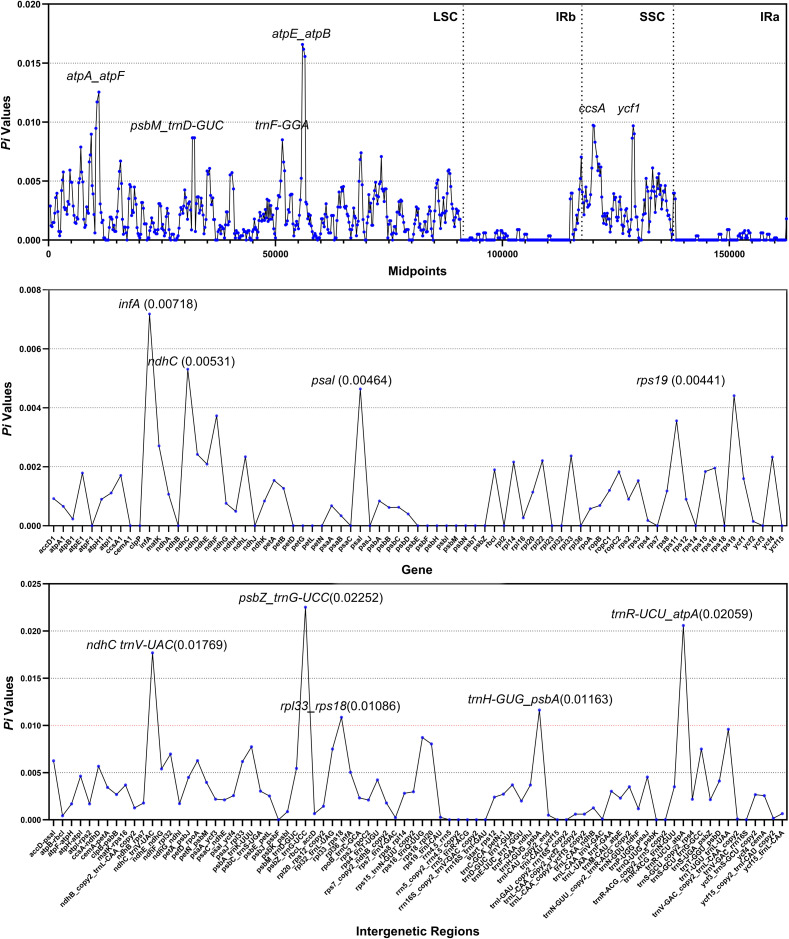
Comparative analysis of nucleotide diversity (*Pi*). The horizontal coordinates indicate the midpoint position/gene name/intergenetic region, and the vertical coordinates represent the *Pi* value.

DNA polymorphism analyses were conducted to determine the nucleotide diversity (*Pi*) of the complete chloroplast genome, PCGs, and intergenetic regions (**Figure 6**). Intergenetic regions presented greater nucleotide polymorphisms than the PCG regions did, which was consistent with the whole-genome alignment results among 18 *Crataegus* and *Mespilus* species. The most highly variable regions included four PCGs (*infA*, *ndhC*, *pasl*, and *rps19*) along with five intergenetic regions (*ndhC~trnV-UAC*, *psbZ~trnG-UCC*, *rpl33~rps18*, *trnH-GUG~psbA*, and *trnR-UCU~atpA*), which may be potential molecular markers for the identification of *Crataegus* species.

The Ka, Ks, and Ka/Ks ratios of PCGs in 18 *Crataegus* and *Mespilus* species were analyzed, revealing the evolutionary rates of these species relative to those of *C. kansuensis* (**Supplementary Figure S2**; **Supplementary Table S7**). Overall, most of the *Ka/Ks* ratios of the 42 PCGs were less than 1, indicating that these PCGs were under purifying selection. The *Ka/Ks* ratio of the *rpoC2* gene exceeded more than 1 in *C. monogyna* (2.11), *C. laevigata* (1.83), *C. songarica* (1.83), and *M. germanica* (1.83). The high Ka/Ks ratios for *rpoC2* in these species indicate that they may be phylogenetically distant from other *Crataegus* species. Similarly, *ndhB* presented a high Ka/Ks ratio in *C. crus-galli*, *C. jozana*, *C.× lavalleei*, and *C. phaenopyrum*, which notably clustered together.

### Validation of candidate DNA barcode

Based on the results of DNA polymorphism analyses (**Figure 6**), PhyloSuite was employed to extract the three intergenic regions (*ndhC~trnV-UAC*, *psbZ~trnG-UCC*, and *trnR-UCU~atpA*) with the highest nucleotide diversity from the chloroplast genomes of 44 *Crataegus* and *Mespilus* species. Only the *ndhC~trnV-UAC* sequence was present in all 44 chloroplast genomes. Subsequently, we constructed a phylogenetic tree using the *ndhC~trnV-UAC* sequences (**Supplementary Figure S3B**), and compared it with the phylogenetic tree constructed based on the complete chloroplast genomes (**Supplementary Figure S3A**). The results showed that the clustering results of the *ndhC~trnV-UAC* sequences for the vast majority (87.75%) of *Crataegus* and *Mespilus* species were consistent with those of the complete chloroplast genomes, and both could divide these accessions into four clades. The phylogenetic tree constructed based on the *ndhC~trnV-UAC* sequencing could accurately distinguish the two *Crataegus* subgroups (C. subg. Crataegus and C. subg. Sanguineae) native to China and also clustered the European-native *Crataegus* species into a separate clade (**Supplementary Figure S4**). These results indicated that *ndhC~trnV-UAC* could serve as a candidate DNA barcode for *Crataegus* species identification.

### Phylogenetic and divergence time analysis

Taxonomic analysis of the chloroplast genomes of 18 *Crataegus* and *Mespilus* species, along with 31 published *Crataegus* and related *Amelanchier* species, was conducted to investigate the evolution of *Crataegus* species. Generally, high congruence was observed between the maximum likelihood (ML) and Bayesian inference (BI) trees, and 49 species and variants were divided into four main clades and one outgroup on the basis of their chloroplast genomes (**Supplementary Figure S5**). *Crataegus* and *Amelanchier* were separated into two groups. For the genus *Crataegus*, 44 species and variants were divided into four distinct clades. The Chinese *Crataegus* species originating from the northeast (*C. sanguinea, C. dahurica*, and *C. maximowiczii*), central (*C. wilsonii, C. aurantia*) and western regions (*C. altaica, C. kansuensis*) were within Clade I, which were belong to C. subg. Sanguineae. C. subg. Americanae plants were within Clade II. These two clusters originated from a common ancestor. *C*. *pinnatifida* and *C*. *pinnatifida* Bge. var. *major* formed Clade III with *Crataegus* species originating from the southwest (*C*. *scabrifolia*), and central regions (*C*. *hupehensis, C. shensiensis*, and *C. cuneata*) of China. *C. songarica* and European *Crataegus* plants were within Clade IV. All species in Clade III and Clade IV were belong to C. subg. Crataegus and C. subg. Mespilus (L.), which originated from a common ancestor. *Crataegus* spp. plants (GSSZ and ZWSZ) may represent the independent species of *Crataegus* similar to *C. wilsonii* and *C. kansuensis. Crataegus* spp. plants (JRY, RR2H, and RR3H) belong to *C*. *pinnatifida*.

**Figure 7:**
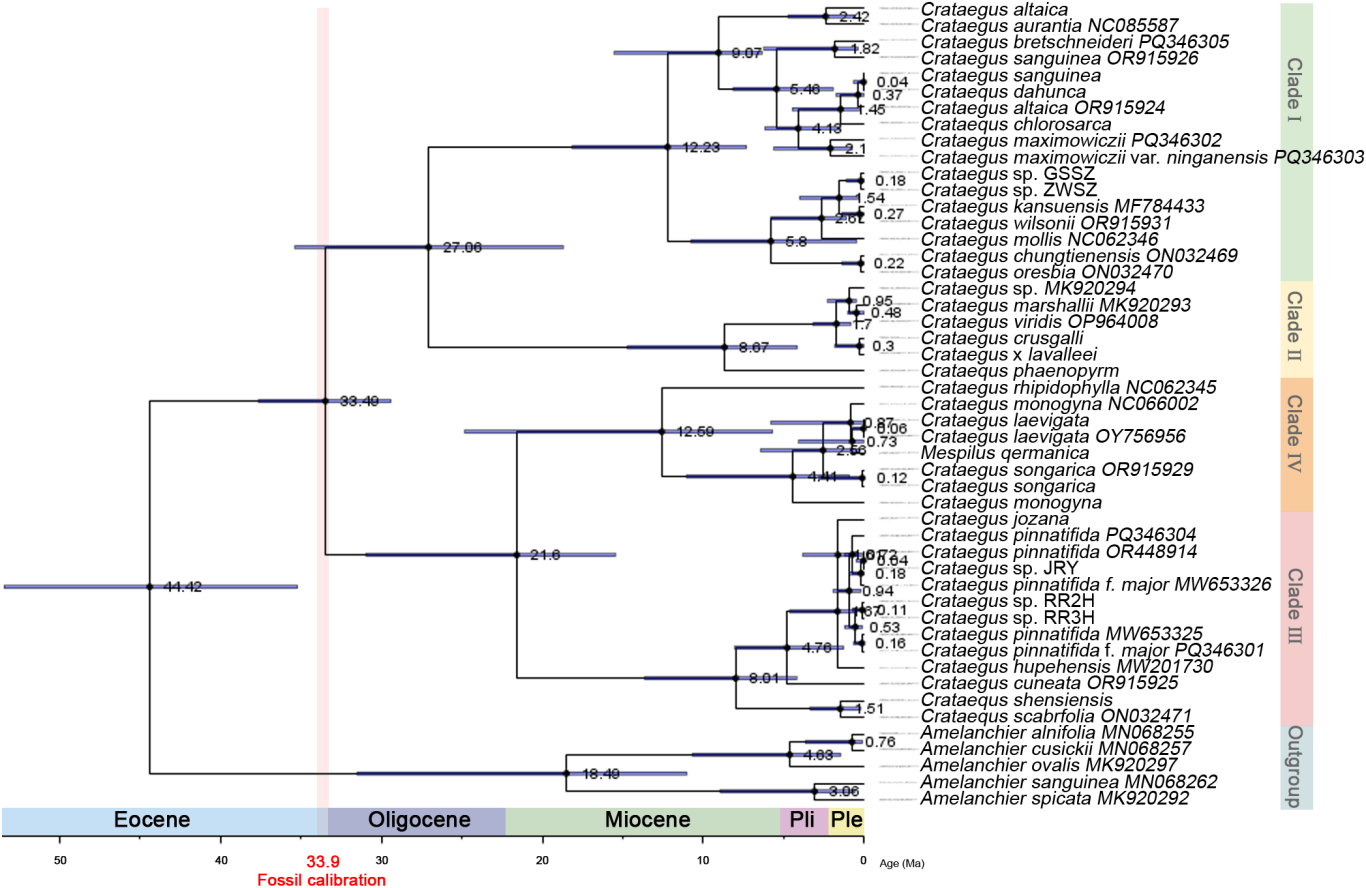
Divergence time estimation for *Crataegus*, *Mespilus* and *Amelanchier* on the basis of complete chloroplast genomes. The number at each node represents the median divergence time, and the node bars represent the 95% HPD (highest posterior density). The ruler on the lower left represents the geologic timescale. Eocene (33.90 ~ 55.80 Ma); Oligocene (23.03 ~ 33.90 Ma); Miocene (5.33 ~ 23.03 Ma); Pli (Pliocene, 1.81 ~ 5.33 Ma); Ple (Pleistocene, 0.01 ~ 1.81 Ma).

The divergence time of *Crataegus* species was estimated (**Figure 7**). The divergence clades of these genera were consistent with the phylogenetic trees, and the *Crataegus* and the outgroup were expected to differentiate 44.42 Ma (Eocene). The differentiation between Clades I + II and III + IV was estimated to have occurred at approximately 33.49 Ma (Oligocene). The divergence time between some *Crataegus* species from Northeast China and their North American congeners was 27.06 Ma. *C. phaenopyrum* and other North American *Crataegus* species differentiated approximately 8.67 Ma in Clade II. For the other group of Chinese *Crataegus* species, their divergence time from European *Crataegus* species was estimated at 21.06 Ma. *Crataegus rhipidophylla* and other European *Crataegus* species differentiated approximately 12.59 Ma. The divergence between *M. germanica* and *C. laevigata* occurred approximately 0.88 Ma.

### Ancestral area reconstruction analysis

**Figure 8:**
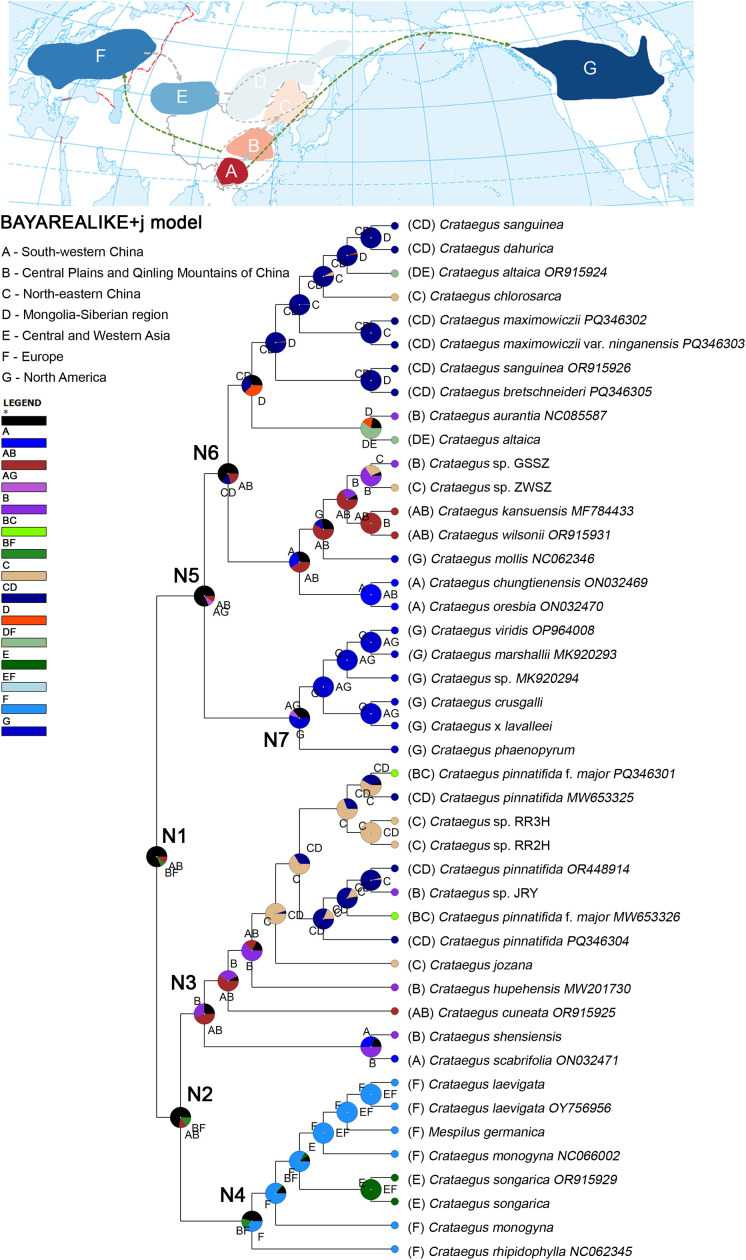
Biogeographic reconstruction in RASP utilizing the BAYAREALIKE + j model, based on the complete chloroplast genomes phylogenies obtained from BEAST analysis. Pie charts depict the most likely distribution locations for the most recent common ancestor. Other ancestral ranges are represented in black and marked with an asterisk.

The BioGeoBEARS analyses in RASP identified that BAYAREALIKE + j was the best-fit biogeographical model with highest AICc_wt value among the six models for chloroplast genomes of *Crataegus* and *Mespilus* (**Supplementary Table S4**). Therefore, we have presented the reconstruction result of BioGeoBEARS with BAYAREALIKE + j DEC model (**Figure 8**). The most recent common ancestor of the entire *Crataegus* clade at Node 1 had a core distribution spanning South-western China (A), the Central Plains and Qinling Mountains of China (B), and Europe (F), providing compelling evidence for a broad trans-Eurasian distribution of this genus during its early evolutionary radiation. Its evolutionary dynamics fall into three distinct biogeographic pathways.

Specifically, the East Asian endemic pathway (Nodes 1, 2, 3, 6) reflects long-term *in-situ* diversification and intra-regional dispersal within the putative East Asian cradle. Node 2 retained the ancestral range (A+B), laying the groundwork for East Asian endemic clades; Node 3 saw the derived lineage persist in South-western to Central China and undergo adaptive radiation to yield endemic taxa represented by *C. scabrifolia*; Node 6 expanded northeastward from the A–B ancestral range, colonizing North-eastern China (C) and the Mongolian-Siberian region (D) and evolving low-temperature acclimation species (e.g., *C. dahurica*, *C. maximowiczii*).

In parallel, the transcontinental dispersal pathway (Nodes 1, 2, 4) delineates intercontinental expansion from East Asia to Europe and Central Asia. Node 2 generated a subclade with westward dispersal potential into Europe; Node 4 extended its range from region B through Europe (F) to Central Asia (E), driving the emergence of the widespread *C. monogyna* (Europe) and endemic *C. songarica* (Central and Western Asia). On the other hand, the transoceanic dispersal pathway (Nodes 5, 7) illuminates the trans-Pacific dispersal of *Crataegus* from East Asia to North America: Node 5 achieved long-distance dispersal from South-western China (A) to North America (G) via the Bering Land Bridge—a key biogeographic corridor—to establish the founder population of the North American clade, while Node 7 underwent independent diversification in North America to produce endemic taxa such as *C. phaenopyrum*.

## Discussion

With minor variations, the whole chloroplast genome is mostly conserved in terms of its quadripartite structure, size, number and placement of genes, and GC content (Daniell et al., 2016). Typical quadripartite structures containing LSC, SSC, and IR sections were found in the newly sequenced chloroplast genomes of 18 *Crataegus* and *Mespilus* (**Figure 2**). These genome structures resembled those of previously identified *Crataegus* species (Wu et al., 2022; Zhang et al., 2022; Meng et al., 2025). The chloroplast genomes of vegetable species are 120~160 kb in length, whereas those of fruit species are 140~180 kb in length (Daniell et al., 2021). The chloroplast genomes in this study were similar in length and conserved; they ranged from 159,638 bp in *Crataegus* sp. (RR2H, RR3H) to 159,973 bp in *C. phaenopyrum* (**Table 1**). Most land plant plastid genomes contain 110~130 genes, over 80 of which encode proteins involved in photosynthesis and other processes (Daniell et al., 2021). Eighteen *Crataegus* and *Mespilus* chloroplast genomes encoded 119~132 genes. The LSC regions were 87,665~88,081 bp in length, and the SSC regions were 19,139~19,295 bp in length. The pair of inverted IRa/IRb regions was 26,311~26,396 bp in length (**Table 1**) With GC levels and compositions ranging from 36.59% to 36.65%, the *Crataegus* and *Mespilus* species were found to be relatively conserved.

Two large inverted repeats (IRs) represent a defining feature of chloroplast genomes (Lin et al., 2012). As a key utility of this structural element, variations in IRs length not only serve as a reliable marker for phylogenetic analysis but also correlate with changes in chloroplast genome size (Wolf et al., 2003). In fact, dynamic expansion and contraction of IR regions have been documented across diverse land plant lineages (Wang et al., 2008; Zhu et al., 2016), with a notable example being the significant IR depletion observed in 13 species belonging to the *Papilionoideae* subfamily (Qin et al., 2025). According to previous studies, the IRs length of *Crataegus* species exhibits minimal variation (Wu et al., 2022; Zhang et al., 2022). Consistent with these findings, our results demonstrated that the IR region length across the eighteen chloroplast genomes showed no significant differences, ranging from 26,331 to 26,396 bp. Two distinct types of genes were identified in the IR regions and their adjacent border areas: specifically, *rpl2* was localized within either the IRb or IRa region, while *ycf1* was situated precisely at the junction between the small single-copy (SSC) region and the IRa region (**Figure 3**). Therefore, the degree of similarity in the inverted repeat (IR) region reflects the conservation of the chloroplast genome in the genus *Crataegus*.

Repetitive DNA sequences (repeats) are patterns of nucleic acids that exist in many copies throughout the genome (Biscotti et al., 2015). Repetitive DNA sequences perform important functions in promoting evolution, causing diversity, and regulating gene expression (Liao et al., 2023). Sequence arrangement and evolution of the chloroplast genome are more influenced by larger and more complex repeat sequences (Huang et al., 2014). In 18 newly sequenced and 31 published chloroplast genomes of *Crataegus*, *Mespilus* and *Amelanchier*, we identified three different kinds of repetitive sequences (**Figure 4**). The quantity and location of scattered repetitions among the genomes varied significantly. The greatest number of forward and palindromic repeats were found in *C. monogyna* (36) and *C. pinnatifida* f. *major* (30), respectively. Chloroplast genomes vary greatly in terms of SSR copy number diversity, which could be used as molecular markers for species identification and population genetics (Li et al., 2020; Ping et al., 2021; Wang et al., 2021). In this study, we identified 74~87 SSRs within 49 chloroplast genomes, respectively (**Supplementary Table S6**). The mono-nucleotides (P1) were the most abundant SSRs in each genome, ranging in quantity from 41 (*C. marshallii*, MK920293) to 56 (*A. cusickii*, MN068257, *A. ovalis*, MK920297). These SSR markers hold considerable potential as tools for species identification and phylogenetic analysis of *Crataegus*.

The creation of mutational hotspots for interspecies discriminating and species-level phylogenetic analysis is facilitated by multi-genome alignments (Liu et al., 2019; Abdullah et al., 2020; Wang et al., 2021; Kłubowicz et al., 2024). To resolve phylogenetic issues in different plant species, a number of plastid DNA markers obtained from highly variable coding and noncoding regions may be employed. Prior successes have been reported with the coding gene *ndhA* in *Hordeum* (Yuan et al., 2023), the noncoding region near *rbcL* in the grass family (Morton and Clegg, 1993), and the intergenetic sequence *rpoC2-rps2* in *Artemisia* (Shahzadi et al., 2020). In this study, we determined the nucleotide diversity (*Pi*) of the complete chloroplast genome, protein coding genes (PCGs) and intergenetic regions among 18 *Crataegus* and *Mespilus* species. Four PCGs (*infA*, *ndhC*, *pasl*, and *rps19*) along with five spacer sequences (*ndhC~trnV-UAC*, *psbZ~trnG-UCC*, *rpl33~rps18*, *trnH-GUG~psbA*, and *trnR-UCU~atpA*) showed high variation (**Figure 6**). The phylogenetic tree constructed based on the *ndhC~trnV-UAC* sequence displayed a branching topology that is largely consistent with that of the tree built using the complete chloroplast genome. The inconsistent distribution of six accessions in the two phylogenetic trees is precisely due to the selective use of variant nucleotide sequences (**Supplementary Figure S3**). Validated by our experimental results, *ndhC~trnV-UAC* could serve as an effective DNA barcode for the identification of *Crataegus* species, especially for distinguishing the plants of the two *Crataegus* subgroups native to China (**Supplementary Figure S4**).

The Ka/Ks ratio serves as a critical metric for deciphering evolutionary pressures acting on genes. In this study, we calculated the Ka/Ks values of 42 protein-coding genes (PCGs) across 18 *Crataegus* and *Mespilus* species (**Supplementary Table S7**). Overall, the majority of PCGs exhibited significantly low Ka/Ks ratios (< 1) indicating that these genes are primarily under purifying selection and thus relatively conserved in sequence. Notably, the *rpoC2* gene displayed the highest Ka/Ks ratio (> 1), reflecting non-synonymous mutations in this gene were positively selected. As a member of the RNA polymerase subunit gene family, *rpoC2*, together with *rpoB* and *rpoC1*, encodes the β″, β, and β′ subunits of RNA polymerase, respectively—key components for maintaining the semiautonomous function of chloroplasts (Börner et al., 2015). The *rpoC2* gene in *C. monogyna*, *C. laevigata*, *C. songarica*, and *M. germanica* exhibits high Ka/Ks ratios (Ka/Ks > 1) and clusters as a single clade in the phylogenetic tree (**Supplementary Figures S2**, **S5**). These reflected the adaptive evolutionary characteristics of these four species, which have undergone advantageous non-synonymous mutations driven by positive selection to cope with similar environmental pressures. A similar pattern was observed in a single clade of phylogenetic tree: *C. crus-galli*, *C. jozana*, *C. × lavalleei*, and *C. phaenopyrum*, which all exhibit a high Ka/Ks ratio in the *ndhB* gene.

It has been difficult to determine phylogenetic relationships in Rosaceae because of apomixis, frequent hybridization, and intricate historical diversification (Xue et al., 2019). The genomes of chloroplasts display typical traits of maternal inheritance. Our results revealed that Clades I and II which include the C. subg. Sanguineae and C. subg. Americanae species were closely related (**Supplementary Figure S5**). These results support Phipps’s hypothesis (Phipps, 1983) that Eurasian and North American *Crataegus* species exchange genes across the Bering Strait. The genus *Mespilus*, which belongs to the C. subg. Mespilus (L.), has two widely distributed species, *M. germanica* L. and *M. canescens* J.B. Phipps. The natural distribution range of *M. germanica* L. is concentrated in the Europe of the Caspian–Black Sea coast (Popović-Djordjević et al., 2023). Since Linnaeus, academic taxonomy has largely maintained *Mespilus* and *Crataegus* separately, but not necessarily for adequate reasons. *Mespilus* and *Crataegus* differ in morphology, which is why Phipps argued for the retention of a monotypic *Mespilus* (Phipps, 2016). However, according to the phylogenetic analyses results of intergenetic cpDNA regions and nuclear sequences, *Mespilus* and *Crataegus* are related genera that are members of the Rosaceae tribe *Pyrea* (Lo et al., 2007; Talent et al., 2008). Phylogenetic analysis (**Supplementary Figure S5**) further confirmed a close phylogenetic relationship among *C. monogyna*, *C. laevigata*, and *M. germanica* in the present study.

With the rapid development of high-throughput sequencing technology, chloroplast genomes have been widely applied as super-barcodes, which can provide effective information for resolving phylogenetic relationships and identifying plants (Liu et al., 2025; Shi et al., 2025). In our previous study, although four *Crataegus* individuals (GSSZ, ZWSZ, RR3H, RR5H) were classified as *C. pinnatifida*, their morphological characteristics of fruits and leaves exhibited certain differences from those of typical *C. pinnatifida* (**Figure 1**). Furthermore, the phylogenetic tree constructed based on specific locus amplified fragment sequencing (SLAF-seq) clustered GSSZ and ZWSZ into a separate clade; RR3H and RR5H into another distinct clade. These two clades showed a relatively distant genetic relationship with *C. pinnatifida* (Du et al., 2019). Our results indicated that RR2H and RR3H share the closest genetic relationship with *C. pinnatifida* (**Supplementary Figure S5**), confirming that these two *Crataegus* individuals are either *C. pinnatifida* or its varieties. GSSZ and ZWSZ belonging to C. subg. Sanguineae are most closely related to *C. kansuensis* and *C. wilsonii* (**Supplementary Figure S5**). These findings also explained the phenotypic similarity among GSSZ, ZWSZ, and *C. kansuensis*.

The geographic origin of *Crataegus* species remains controversial. Phipps proposed that the origin regions of *Crataegus* was East Asia or South America. The original population of *C. scabrifolia* spread westward to the European continent and on the other hand, evolved northward into most Asian species, including *C. sanguineae*, *C. hupehensis*, and *C. pinnatifida*, while crossing the Bering Strait to evolve into most existing North American hawthorn species (Phipps, 1983). Based on ITS sequences, cpDNA fragments, and *LEAFY* gene data, Lo et al. (2009) analyzed 37 *Crataegus* accessions sampled from East Asia, northwestern North America, northeastern North America, and Europe, and proposed that the genus *Crataegus* originated in northeastern North America and Europe, with *C. germanica* and *C. brachyacantha* identified as its ancestral species. However, *Crataegus* originating from southwestern China were not employed in Lo et al.’s research. Subsequent relevant study indicated that southwestern Chinese *Crataegus* share a gene pool with European lineages, while northeastern Chinese populations probably originated from North American species (Du et al., 2019). Recently, the plastome-based research indicate that East Asian *Crataegus* species migrated transoceanically via the Bering Land Bridge to form North American taxa, while the westward dispersal of *C. songarica* ancestors promoted the evolution of European lineages (Meng et al., 2025). Our biogeographic and molecular dating analyses reveal that the ancestral clade of *Crataegus* was widely distributed across eastern and western Eurasia (**Figure 8**). A comprehensive synthesis of prior studies and our novel data further confirms China as one of the genus’ primary centers of origin. The evolutionary trajectories of *Crataegus* elucidated herein, which are characterized by two major dispersal events (transcontinental expansion from East Asia to Europe and Central Asia, and trans-Pacific migration from East Asia to North America), are highly congruent with the findings of Phillips and Meng while providing empirical support for Du’s hypothesis. Notably, while our study differs from that of Meng et al. (2025) in the sampling size of *Crataegus* species and geographic partitioning methodology, the latter proposed that the ancestral lineage of *C. songarica* facilitated the formation of European *Crataegus* species. In contrast, our plastome-based analyses incorporating a larger number of European *Crataegus* individuals demonstrate that the formation of European *Crataegus* species predated that of *C. songarica*. Collectively, these results clarify the spatiotemporal dynamics underlying the global diversification of *Crataegus*, laying a robust foundation for future investigations into the genus’ adaptive evolution in response to historical climatic fluctuations.

## Conclusion

In the present study, 18 complete chloroplast genomes from *Crataegus* and *Mespilus* species were assembled and compared. Numerous facets of the chloroplast genome, such as repeat sequence and microsatellite assays, gene annotation, and codon use studies have been investigated. Regions such as *ycf2*, *rrn23*, and *rrn16* presented the highest degree of conservation among the exon and UTR regions. Nonetheless, *ndhC~trnV-UAC* demonstrated significant variability among the chloroplast genomes, and could be regarded as potential molecular markers for further phylogenetic assessments. Maximum likelihood and Bayesian phylogenetic trees revealed genetic relationships among *Crataegus* and *Mespilus* species, and confirmed the taxonomic status of *Crataegus* individuals (GSSZ, JRY, RR2H, RR3H, ZWSZ). The results of divergence time showed that the crown age of C. subg. Crataegus was about 33.487 Ma, and then started to diverge into the C. subg. Americanae and C. subg. Sanguineae around 27.059 Ma. Based on the results of molecular evidence, we speculate that China represents a putative maternal origin of *Crataegus* species. This study not only enriched the complete chloroplast genome resources of *Crataegus*, but also provided useful information for further studies of the evolution and phylogeny of *Crataegus* species.

## Data Availability

The original data presented in the article has been successfully deposited in the following database: NCBI, PX676094-PX676143.
