# Supplementary figures and images for: Comparative genomic and phylogenetic analyses of Crataegus chloroplast genomes: insights for evolution and identification
Source: Front Plant Sci. 2026 Feb 11;17:1767012. doi: 10.3389/fpls.2026.1767012 (PMC12932471; doi:10.3389/fpls.2026.1767012)

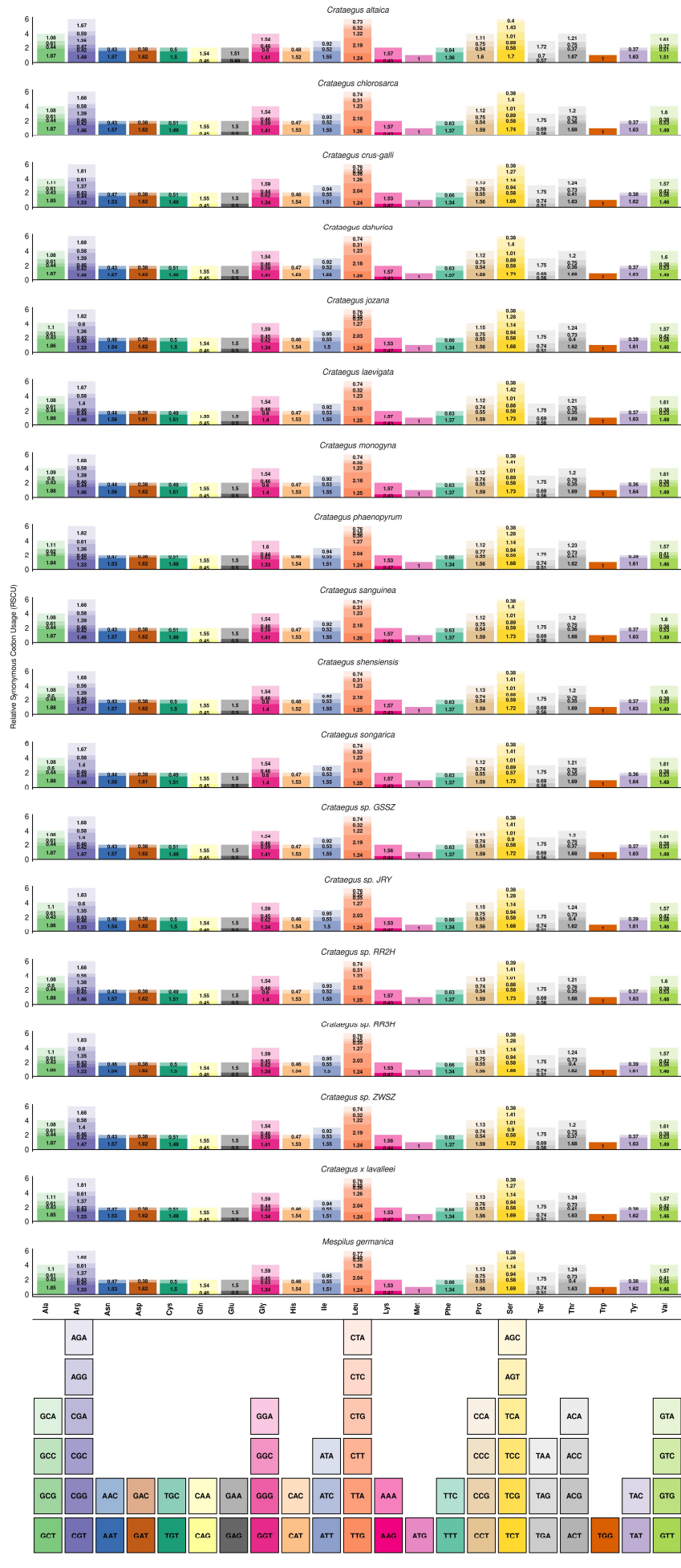

Figure S1 Relative synonymous codon usage (RSCU) values of 18 chloroplast genomes

Supplement: Supplementary file 1 [file DataSheet1.pdf]
