# Supplementary material for: Comparative genomic and phylogenetic analyses of Crataegus chloroplast genomes: insights for evolution and identification
Source: Front Plant Sci. 2026 Feb 11;17:1767012. doi: 10.3389/fpls.2026.1767012 (PMC12932471; doi:10.3389/fpls.2026.1767012)
Supplement: Supplementary file 2 [file DataSheet2.pdf]

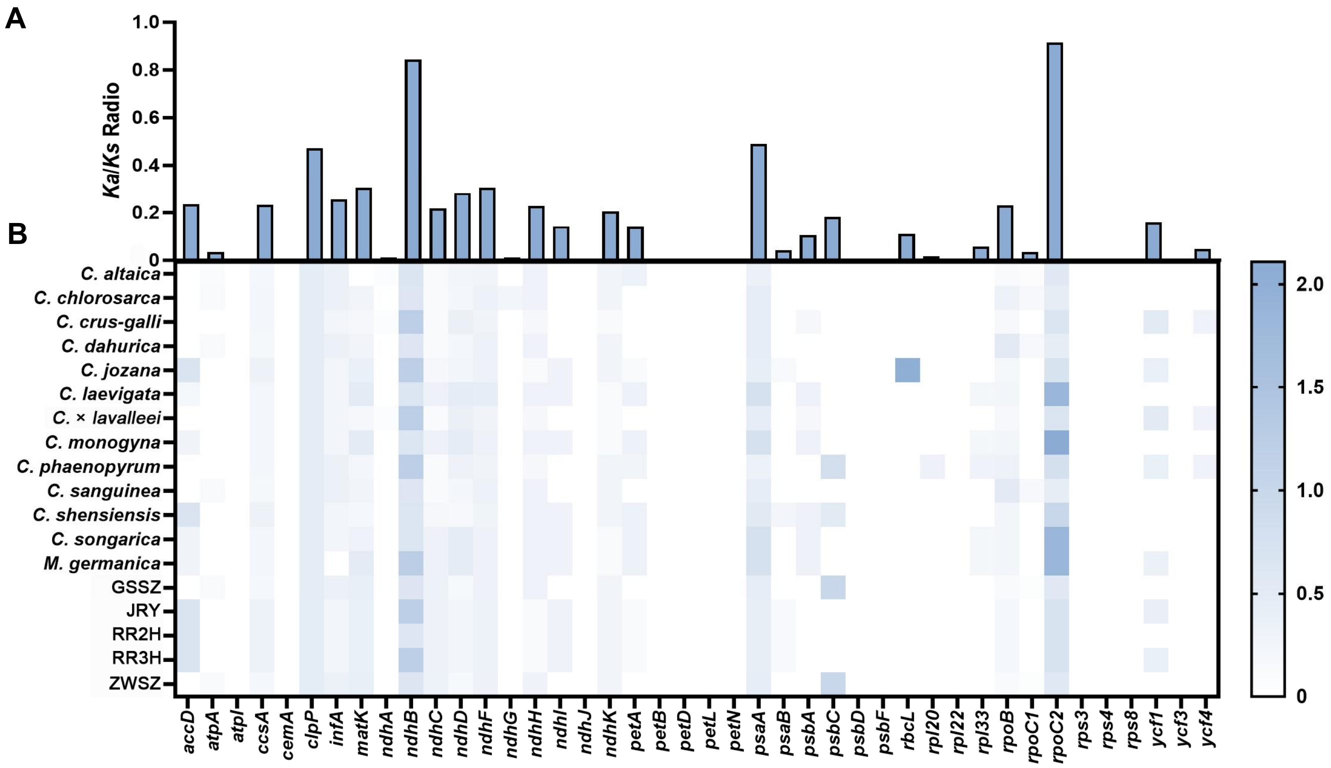

Figure S2 Results of the selective pressure analysis. (A) A bar chart showing the average Ka/Ks ratios for 42 PCGs sequences across species. (B) Heatmap of the 42 PCGs sequences across 18 individuals normalized with simple linear interpolation, where the depth of color represents purifying selection or positive selection, with a baseline at Ka/Ks=1
