# Supplementary material for: Comparative genomic and phylogenetic analyses of Crataegus chloroplast genomes: insights for evolution and identification
Source: Front Plant Sci. 2026 Feb 11;17:1767012. doi: 10.3389/fpls.2026.1767012 (PMC12932471; doi:10.3389/fpls.2026.1767012)
Supplement: Supplementary file 3 [file DataSheet3.pdf]

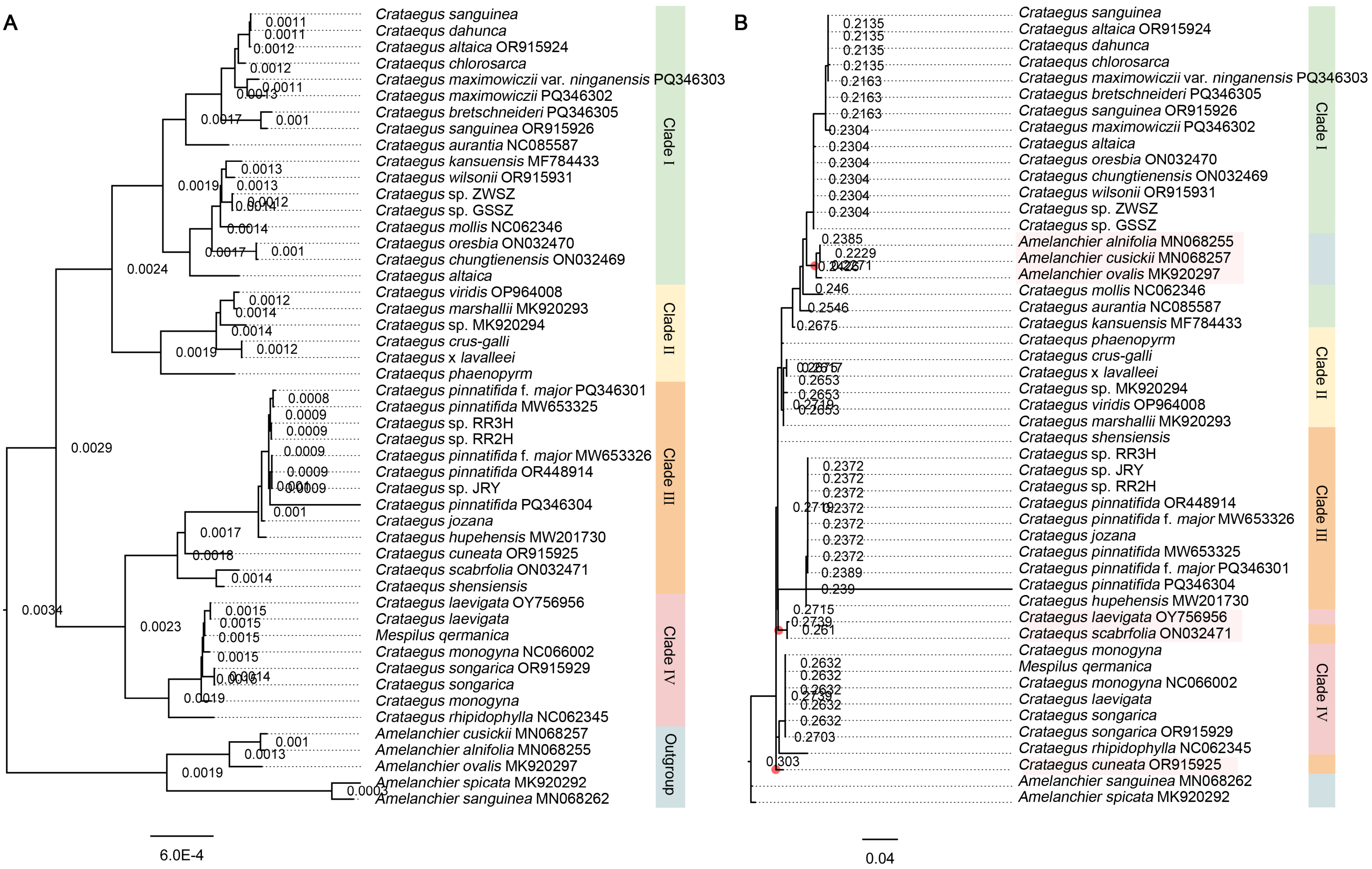

Figure S3 ML phylogenetic trees based on complete chloroplast genomes (A) and *ndhC~trnV-UAC* sequences (B) with *Amelanchier* as outgroups. Node ages values are displayed on the Node. The color represents the different subgroups of *Crataegus*.
