# Supplementary material for: Comparative genomic and phylogenetic analyses of Crataegus chloroplast genomes: insights for evolution and identification
Source: Front Plant Sci. 2026 Feb 11;17:1767012. doi: 10.3389/fpls.2026.1767012 (PMC12932471; doi:10.3389/fpls.2026.1767012)
Supplement: Supplementary file 4 [file DataSheet4.pdf]

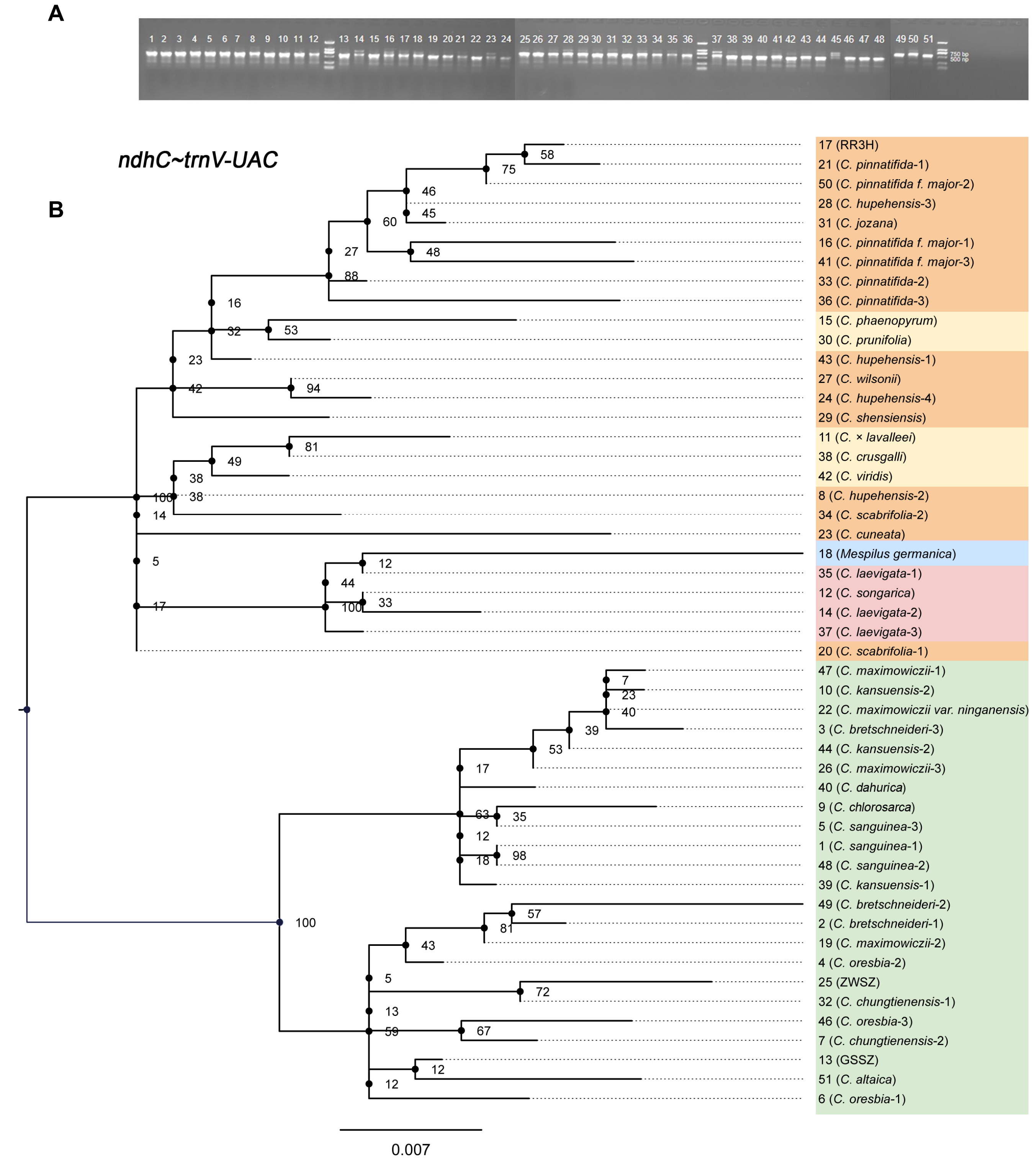

Figure S4 Validation of candidate DNA barcode. (A) *ndhC~trnV-UAC* sequencing results of *Crataegus* individuals; (B) Phylogenetic trees of *Crataegus* individuals using maximum likelihood (ML) based on *ndhC~trnV-UAC* intergenetic spacer sequences. The color represents the different subgroups of *Crataegus*. Red and orange: C. subg. Crataegus; Yellow: C. subg. Americanae; Green: C. subg. Sanguineae; Blue: C. subg. Mespilus (L.)
