# Supplementary material for: Comparative genomic and phylogenetic analyses of Crataegus chloroplast genomes: insights for evolution and identification
Source: Front Plant Sci. 2026 Feb 11;17:1767012. doi: 10.3389/fpls.2026.1767012 (PMC12932471; doi:10.3389/fpls.2026.1767012)
Supplement: Supplementary file 5 [file DataSheet5.pdf]

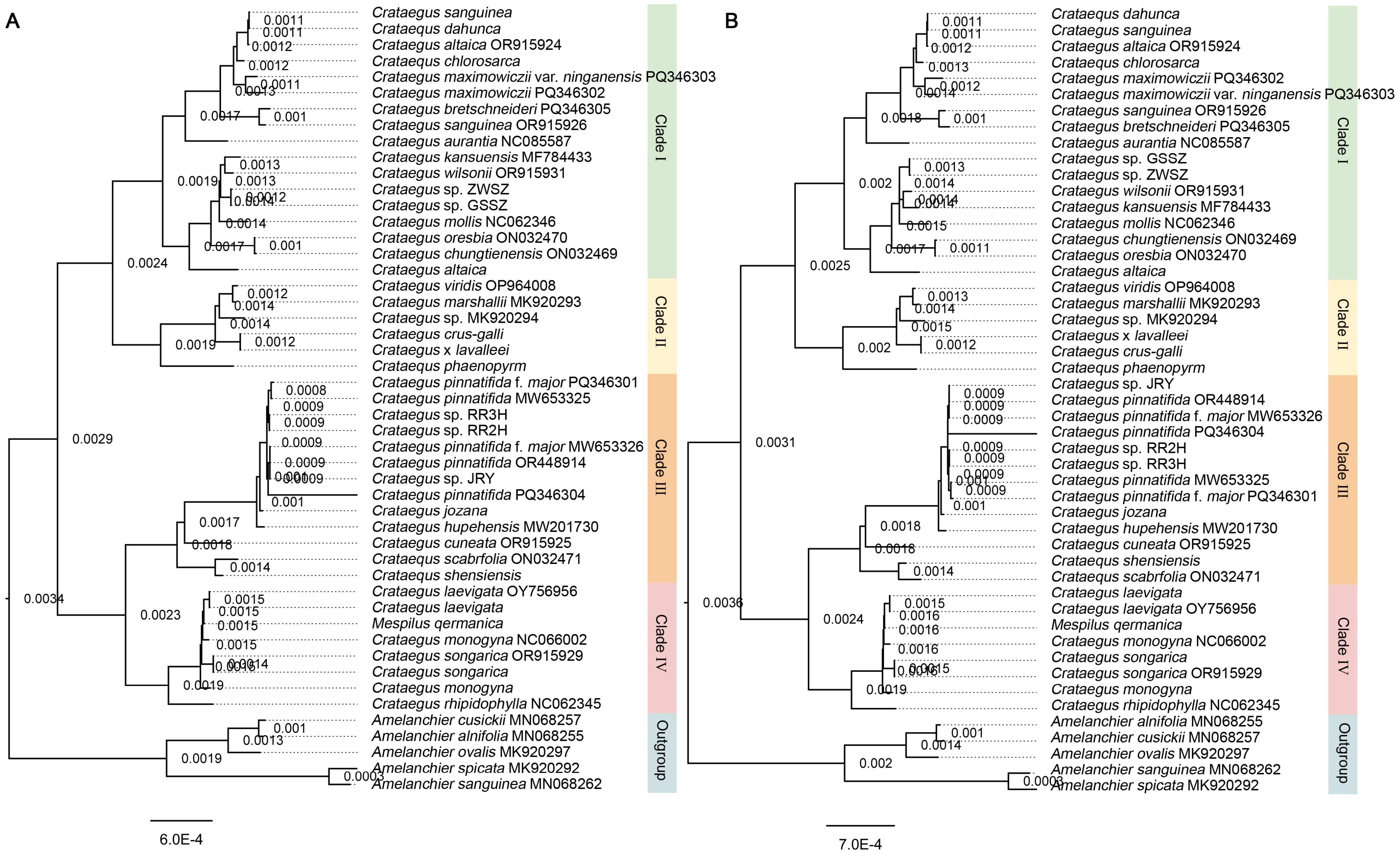

Figure S5 ML (A) and BI (B) phylogenetic trees based on complete chloroplast genomes with *Amelanchier* as outgroups. Node ages values are displayed on the Node. The color represents the different subgroups of *Crataegus*. Clade I: C. subg. Sanguineae; Clade II: C. subg. Americanae; Clade III and Clade IV: C. subg. Crataegus, C. subg. Mespilus (L.)
